# Recommendations for diagnosis and treatment of Atypical Hemolytic Uremic Syndrome (aHUS): an expert consensus statement from the Rare Diseases Committee of the Brazilian Society of Nephrology (COMDORA-SBN)

**DOI:** 10.1590/2175-8239-JBN-2024-0087en

**Published:** 2025-02-07

**Authors:** Maria Helena Vaisbich, Luis Gustavo Modelli de Andrade, Maria Izabel Neves de Holanda Barbosa, Maria Cristina Ribeiro de Castro, Silvana Maria Carvalho Miranda, Carlos Eduardo Poli-de-Figueiredo, Stanley de Almeida Araujo, Miguel Ernandes, Maria Goretti Moreira Guimarães Penido, Roberta Mendes Lima Sobral, Oreste Ferra, Precil Diego Miranda de Menezes Neves, Cassiano Augusto Braga da Silva, Fellype Carvalho Barreto, Igor Gouveia Pietrobom, Lilian Monteiro Pereira Palma

**Affiliations:** 1 Universidade Federal de São Paulo, Instituto da Criança, Hospital das Clínicas - HCFMUSP, São Paulo, SP, Brazil.; 2 Universidade Estadual Paulista, Departamento de Medicina Interna, São Paulo, SP, Brazil.; 3 Hospital Federal de Bonsucesso, Serviço de Nefrologia e Transplante Renal, Rio de Janeiro, RJ, Brazil.; 4 Universidade de São Paulo, Unidade de Transplante Renal, São Paulo, SP, Brazil.; 5 Hospital Santa Casa de Belo Horizonte, Belo Horizonte, MG, Brazil.; 6 Pontifícia Universidade Católica do Rio Grande do Sul, Porto Alegre, RS, Brazil.; 7 Universidade Federal de Minas Gerais, Instituto de Nefropatologia, Centro de Microscopia Eletrônica, Belo Horizonte, MG, Brazil.; 8 Hospital Beneficiência Portuguesa de São Paulo, São Paulo, SP, Brazil.; 9 Universidade Federal de Minas Gerais, Nefrologia Pediátrica, Centro de Nefrologia da Santa Casa de Belo Horizonte, Belo Horizonte, MG, Brazil.; 10 Universidade Federal da Bahia, Hospital Universitário Prof. Edgard Santos, Unidade do Aparelho Urinário, Salvador, BA, Brazil.; 11 Universidade Federal de Mato Grosso do Sul, Hospital Universitário Maria Aparecida Pedrossian, Campo Grande, MS, Brazil.; 12 Universidade de São Paulo, Departamento de Nefrologia, São Paulo, SP, Brazil.; 13 Hospital Alemão Oswaldo Cruz, Centro de Diálise e Nefrologia, São Paulo, SP, Brazil.; 14 Clínica Senhor do Bonfim, Departamento de Nefrologia, Feira de Santana, BA, Brazil.; 15 Universidade Federal do Paraná, Curitiba, PR, Brazil.; 16 Universidade Federal de São Paulo, São Paulo, SP, Brazil.; 17 Universidade Estadual de Campinas, Nefrologia Pediátrica, Campinas, SP, Brazil.

**Keywords:** Guidelines, Diagnosis, Treatment, Atypical Hemolytic Uremic Syndrome, Thrombotic Microangiopathy

## Abstract

Atypical hemolytic uremic syndrome (aHUS) is a rare cause of thrombotic microangiopathy (TMA) caused by the dysregulation of the alternative complement pathway. The diagnosis of TMA is made clinically by the triad: microangiopathic hemolytic anemia, thrombocytopenia, and organ damage (mainly acute kidney injury). The heterogeneity of clinical manifestation and the lack of a gold standard diagnostic test makes the precise diagnosis of aHUS a challenging process that may impact patient management. Until one decade ago, there was no specific treatment for aHUS and patients were submitted to plasma therapy (plasma exchange and/or plasma infusion) and/or liver transplantation, procedures that are not free of serious complications and that do not address the underlying pathophysiology of the disease. Since 2011, an anti-C5 complement monoclonal antibody has been approved by the Food and Drug Administration (FDA) for aHUS patients beginning a new era in treatment. Clinical trials on new complement inhibitors may also add to the treatment portfolio in the future. The Brazilian population is a mixed race with a unique genetic and clinical profile. This consensus aims to offer recommendations for the diagnosis and treatment of patients with aHUS in this population based on expert experience, data from the aHUS Brazilian Registry and literature review. The GRADE system was used to classify the quality of the evidence.

## Introduction

Atypical hemolytic uremic syndrome (aHUS) is an ultra-rare cause of thrombotic microangiopathy (TMA), characterized by non-immune hemolytic anemia, thrombocytopenia, and systemic manifestations including renal involvement, frequently manifested as acute kidney injury (AKI). Typically, an abnormality in the regulatory proteins of the alternative complement pathway leads to an excessive formation of the membrane attack complex (C5b-9), causing endothelial cell damage and microthrombi formation throughout the body^1^. Disease-related variants in complement regulatory genes or presence of complement Factor H (CFH) autoantibodies are found in 60–70% of patients^2^. While there is a shift towards using the term complement-mediated HUS, we chose to adhere to aHUS in this consensus, as defined in pivotal trials of complement inhibitors and by the Food and Drug Administration (FDA).

The epidemiology of aHUS is influenced by genetic background and population traits^3,4^. Global data is limited due to the rarity of aHUS. A 2020 systematic review provided initial consistent epidemiological insights^5^. Data from Norway, France, Italy, and Australia estimated the prevalence and incidence of aHUS. Prevalence among individuals aged 20 years or younger ranged from 2.2 to 9.4 per million, with an overall prevalence of 4.9 per million^5^. Annual incidence rates for those older than 20 years varied from 0.26 to 0.75 per million and for all ages from 0.23 to 1.9 per million^5^.

The diverse genetic ancestry of the Brazilian population and its high admixture rate render its population ideal for broadening the genetic spectrum of aHUS^6,7^. The Brazilian aHUS Registry, coordinated by the Rare Diseases Committee of the Brazilian Society of Nephrology (COMDORA-SBN), revealed a unique disease profile^7^. Predominantly affecting women and young adults, a high rate of renal involvement was observed. Pediatric patients had lower hemoglobin and platelet levels on presentation, and higher LDH levels compared to adults. Common genetic variants, notably in the *CFH* gene and a large *CFHR1-3* deletion, were found across age groups^7^, which has implications for the choice of genetic testing methods.

Clinical manifestations depend on the severity of ischemia in affected organs^8^. Associated with the hematological condition, kidney involvement is often observed, manifesting as acute renal lesion, edema, oligoanuria, proteinuria, hematuria, and systemic arterial hypertension. Additionally, there may be central nervous system involvement (mental confusion, lethargy, seizures, coma), gastrointestinal tract disorders (diarrhea, liver disorders, pancreatitis), pulmonary involvement leading to alveolar hemorrhage, ocular complications (amaurosis), cutaneous ischemia (which can lead to necrosis of the extremities), and cardiac involvement^9,10^.

It is important to emphasize that in some cases, a subacute presentation may occur with renal impairment and arterial hypertension with signs of TMA on renal biopsy, but no systemic signs of hemolysis and thrombocytopenia. Therefore, the differential diagnosis of TMA should be considered in any patient presenting kidney injury and low-grade hemolysis (grade 1B)^11^.

aHUS is very heterogeneous in its clinical manifestation, resulting in difficulties in diagnosis and treatment^1^. To address these issues, a group of experts presents the first Brazilian consensus document for the diagnosis and management of patients with aHUS.

Although similar articles have been previously published worldwide, the Brazilian population is unique^7^ and these particularities along with the difficulty of accessing all exams and treatments, justify the development of a national consensus document.

## Methodology

### Goals of the Brazilian Consensus for aHUS

This consensus document was developed as part of an initiative coordinated by COMDORA-SBN to standardize the diagnosis and management of aHUS in Brazil.

A panel of Brazilian experts developed this document based on literature review, data from the aHUS Brazilian Registry, and their own experience with these patients. A meeting was held in São Paulo on August 19 and 20 (2023) to define key points for the document. Literature review was performed on the following databases: PubMed, Scielo, LILACS (Latin American Research Review), and Cochrane Library. The keywords used were: “Atypical Hemolytic Uremic Syndrome” OR “aHUS” AND “Diagnosis” OR “Treatment”. The inclusion criteria used were articles published up to August 2023 in English, Portuguese, or Spanish.

The quality of evidence was determined based on the literature review. In rare diseases, obtaining high-quality evidence is challenging due to the small number of patients and clinical heterogeneity. As randomized controlled trials are scarce, recommendations were derived from systematic reviews, randomized clinical trials, previously published guidelines, case series, cohort studies, and registry data reflecting real-world data^12^. Moreover, meta-analyses of individual trials may help address this issue^13^.

In addition, the personal experience of the panelists was considered, especially in controversial issues. The GRADE system was used to classify the strength of the recommendations and the quality of the evidence (Chart 1)^14,15^.

**Chart 1:** Level of recommendation and quality of evidence

Level of recommendation	
1. Strong Recommendation	Medical and economic benefits are definite.
2. Weak Recommendation	Medical and economic benefits are suggestive of some benefit. The evidence is not sufficient to make a strong recommendation.
Quality of evidence	
A	High-quality evidence: Evidence from a meta-analysis of randomized controlled trials or at least one or more randomized controlled trials.
B	Moderate-quality evidence: Evidence from a randomized controlled study with a serious limitation or large-scale observational studies.
C	Low-quality or very-low-quality evidence: Evidence from small-scale observational studies or non-experimental descriptive studies such as comparative studies, correlations studies, case-control studies, or expert opinions.

Adapted from Cheong *et al., 2016* ^15^.

## AHUS Diagnostic Criteria

### Recognizing Thrombotic Microangiopathy (TMA)

**Figure 1:**
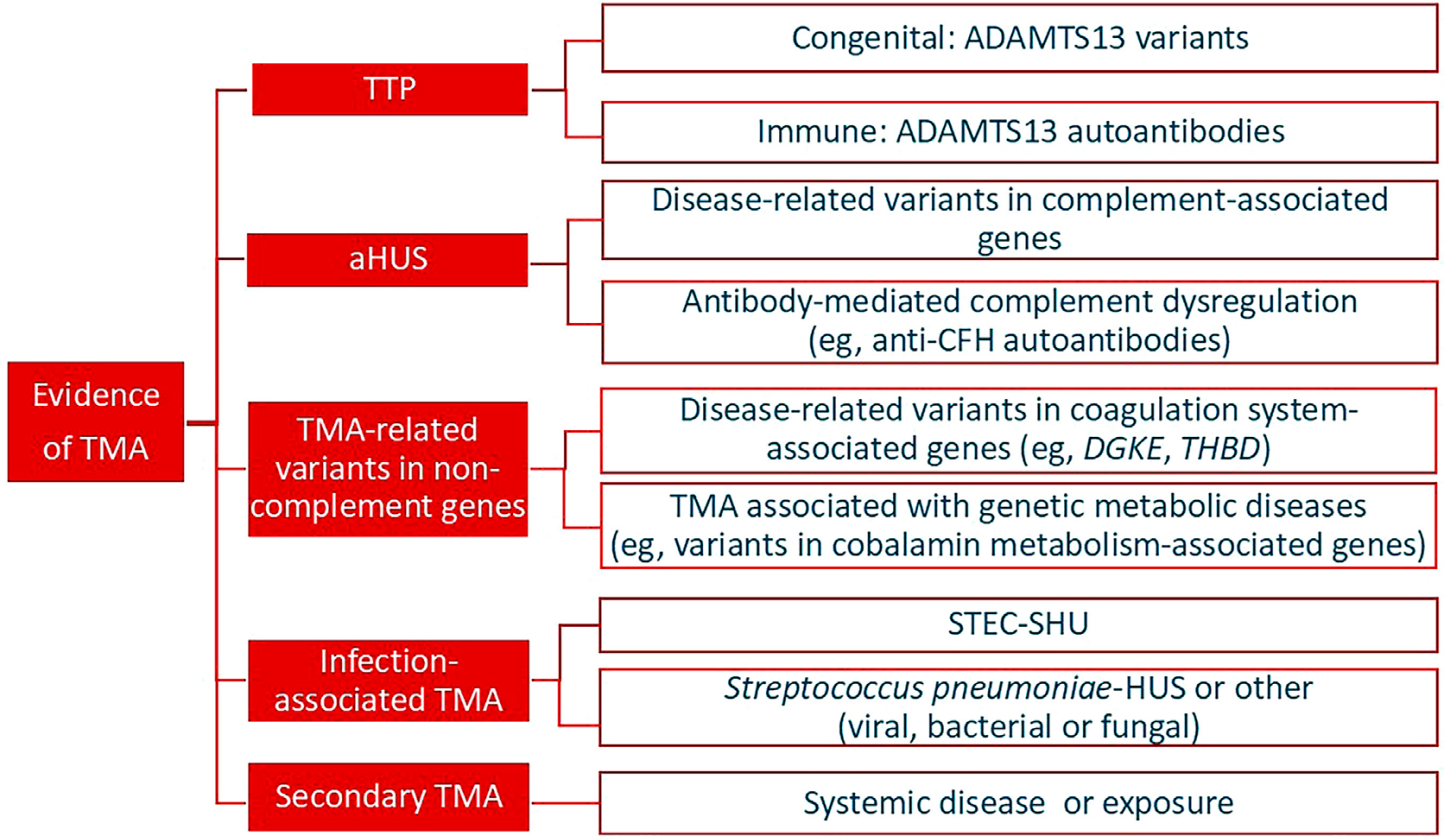
Practical classification of thrombotic microangiopathies. Modified from Genest et al, 2023_17_.

Diagnosis relies on histopathological features, but renal biopsy is often challenging due to thrombocytopenia and severe clinical presentation^16^. The histopathological findings are complex and diverse and can be summarized as shown in Figure 1. Suspected aHUS starts with the TMA triad: microangiopathic hemolytic anemia (MAHA), thrombocytopenia (absolute or signs of progressive platelet consumption), and organ damage (kidneys, heart, brain, gastrointestinal tract, and others)^16^. Renal involvement, observed in all Brazilian aHUS population, is common^7^. This syndrome can manifest at any age, regardless of whether it is inherited or acquired^7^.

It is important to keep in mind that there are some conditions that may mimic TMA, such as prosthetic heart valves or the use of cardiopulmonary bypass^18^, sickle cell crisis in patients with sickle cell anemia, and even emboli of metastatic neoplasia. These conditions can also manifest with MAHA, which are often associated with thrombocytopenia and organ dysfunction, although they are not classified as TMA.

In 2017, the Kidney Disease Improving Global Outcomes (KDIGO) initiative listed all known causes of TMA^19^. Traditionally, TMA is divided into primary and secondary^19^.

**Primary TMA:** The primary causes of TMA have a well-known pathophysiological mechanism and an established treatment. Classically, these include thrombotic thrombocytopenic purpura (TTP) – a severe deficiency of a disintegrin and metalloproteinase with a thrombospondin type 1 motif, member 13 (ADAMTS13, also known as von Willebrand factor-cleaving protease), and aHUS^20^. The other patients with TMA are classified as having secondary TMA.

**Secondary TMA:** Secondary causes of thrombotic microangiopathy typically occur in the context of systemic diseases, and TMA often resolves with treatment or removal of the underlying cause. Classic secondary causes include TMA associated with Shiga toxin (ST) produced by *Escherichia coli* (EC), known as typical hemolytic uremic syndrome (HUS) or STEC-HUS, HUS associated with other infections such as *Streptococcus pneumoniae*-related HUS (*Sp*-related HUS), pregnancy-related TMA, solid organ (especially kidney) and hematopoietic stem cells transplantations, malignancies, autoimmune diseases, drugs, and malignant hypertension^16,20^. They are more frequent than primary TMA. An analysis of 500 patients from four French centers revealed that 94% of cases were secondary to pregnancy (35%), infection (33%), drugs (26%), neoplasia (19%), transplantation (17%), autoimmune diseases (9%), malignant hypertension (4%), and other factors (6%)^20,21^.

The diagnosis of aHUS is only established after ruling out other causes of TMA, such as TTP, STEC-HUS^16^, and secondary TMA conditions^19,22^.

However, as our understanding of TMA advances and underlying mechanisms are elucidated, the classification and nomenclature of TMA continue to evolve. One of the practical schemes suggested by Genest et al.^17^ offers a new TMA classification approach. In the present document, the authors have modified the proposal of Genest et al.^17^ and classify TMA into the following categories:

1) TTP, congenital or acquired; 2) aHUS, a complement-mediated TMA caused by variants in complement-associated genes (congenital) or by antibody-mediated complement dysregulation, such as anti-CFH autoantibodies (auto-immune); 3) TMA associated with variants in non-complement genes, such as those involved in the coagulation system (e.g., *DGKE*, *THBD*) or metabolic defects, such as cobalamin metabolism disturbances; 4) Infection-associated TMA, including STEC-HUS and others; and 5) TMA secondary to systemic disease or drug exposure. This revised classification is illustrated in Figure 1 ^17^.

### Determinig the Etiology of TMA

Once TMA has been identified, the challenge is to establish the correct cause to start a customized treatment immediately. Anamnesis, physical examination, and family health history help identify the etiology of TMA. A positive family history raises the suspicion of a genetic-related disease. Furthermore, recognizing symptoms like those seen in STEC-HUS helps determining the etiology^7^.

The next step is to assess the severity of organ damage, which determines the clinical presentation and is crucial for managing life-threatening situations^8,11^. A systematic approach to identify the underlying cause is essential to reassess targeted therapy^11^.

### Diagnostic Criteria of aHUS

The diagnosis of aHUS is clinical and is established after ruling out other causes of TMA, such as TTP and STEC-HUS, and secondary TMA conditions^16^. Recommendations for diagnostic tests are shown in Chart 2.

**Chart 2:** Recommendations for diagnostic tests

Diagnostic tests	
**Confirm TMA**	Biochemical evaluation
	Hematological exam: hemoglobin, thrombocytes, and reticulocytes
	Serum LDH
	Serum haptoglobin
	Peripheral blood smear (detect the presence of schistocytes)
	Indirect Coombs/direct Coombs
	PT/aPTT/fibrinogen
**Complement testing and other tests/Etiology**	**Detect STEC**
	Stool or rectal swab culture
	PCR for STEC virulence genes in stool
	Serology: serum (*E. coli*) and Yersinia antibodies (anti-LPS antibodies for prevalent serotypes)
	**Detect ADAMTS-13 deficiency**
	Von Willebrand protease activity
	**Test underlying causes (secondary causes)**
	Plasma homocysteine (increased levels are observed in cobalamin disturbances)
	HIV serology, pulmonary cultures, influenza
	ANA/anti dsDNA (Farr)/anti-centromere Ab/antiphospholipid antibodies (anticardiolipin IgG and IgM, anti B/lupus anticoagulant)
	Hemocultures
	Pregnancy testing
	Chest X-ray
	Factor H; factor I antibodies
	Serum CH50
	Serum MAC (C5b-9)
	Serum levels of C3, C4; index C3d/C3
	CD46 expression on leukocytes (poly- or mononuclear leukocytes using a FACS test)
	Blood levels of factor B, factor Bb, C3 convertase, factor H activity, antibodies for factor I, other complement factors and AP50
**Genetic testing/ Etiology**	Complement factor H (*CFH* gene)
	Complement factor I (*CFI* gene)
	Membrane cofactor protein (*MCP* gene)
	Complement factor B (*CFB* gene)
	Complement C3 (*C3* gene)
	Complement factor H-related proteins (*CFHR* genes)
	*CFH*-*CFHR* hybrid gene
	*DGKE* variants (children under 2 years old, especially if nephrotic syndrome is associated)
	Thrombomodulin (*THBD gene*)
	*ADAMTS13* gene: if indicated (PTT)
	*MMACHC* gene: if indicated to exclude defect in cobalamin deficiency (especially patients under 18 years old)
	Other complement genes, if indicated

Abbreviations – TMA: thrombotic microangiopathy; LDH: lactate dehydrogenase; PT: prothrombin time; aPTT: activated partial thromboplastin time; STEC: Shiga toxin-producing Escherichia coli; PCR: polymerase chain reaction; ANA: antinuclear antibody; CH50: measuring the 50% hemolytic complement; MAC: membrane attack complex; AP50: alternative pathway hemolytic complement.

The recommended diagnostic criteria are shown in Chart 3 and Figure 1.

**Chart 3:** Diagnosis criteria of aHUS

Diagnosis criteria	
Kidney biopsy (when available and in the absence of contraindications).	Signs of TMA.
Markers of thrombotic microangiopathy	High LDH, MAHA, thrombocytopenia, schistocytes in peripheral blood smear, low haptoglobin, negative direct Coombs test, high indirect bilirubin.
ADAMTS13 activity	Normal result (above 10%).
PCR for Shiga-toxin or stool culture	Negative result.
Plasma homocysteine level	Normal range result. Note: High level, especially in patients under 18 years old, may be associated with cobalamin metabolism disturbances.

Abbreviations – TMA: thrombotic microangiopathy; LDH: lactate dehydrogenase; MAHA: microangiopathic hemolytic anemia; PCR: polymerase chain reaction.

aHUS is suspected in patients with TMA after ruling out secondary causes, i.e. ADAMTS13 activity is above 10% ruling out TTP and tests for STEC-HUS are negative (grade 1B). Whenever available, complement activation should be investigated based on local resources, although measuring plasma C5b-9 is not yet available in clinical practice. Plasma C3 levels can be assessed - low levels are found in less than 20% of patients and normal levels do not rule out aHUS^23^ (grade 1B). There is still no consensus on complement tests in aHUS.

If ADAMTS-13 activity test is unavailable or while awaiting results, the PLASMIC score is a helpful bedside tool to diagnose TTP, allowing for an early treatment of this lethal disease. The sensitivity and specificity of a PLASMIC score equal or above 6 was 0.85 (confidence interval 0.67–0.94) and 0.89 (95% confidence interval 0.81–0.94)^24^. The PLASMIC score is shown in Table 1 ^25^ and online calculators can be helpful (www.mdcalc.com). Only 5% of patients with TTP present with the classic pentad: fever, hemolytic anemia, thrombocytopenia, neurologic manifestations, and kidney injury.

**Table 1:** PLASMIC score

Parameter	Points
Platelet count <30 x 10^9^/L	1
Hemolysis (reticulocyte count >2.5%, haptoglobin undetectable, or indirect bilirubin >2 mg/dL)	1
No active cancer	1
No history of solid-organ or stem-cell transplant	1
MCV <90fL	1
INR <1.5	1
Creatinine <2 mg/dL	1

Abbreviations – INR: international normalized ratio; MCV: mean corpuscular volume. Adapted from Vyas *et al.,* 2023^25^.

The interpretation of PLASMIC scores is^25^:

- Total points = 0 to 4 – **low** risk for severe ADAMTS-13 deficiency
- Total points= 5 – **intermediate** risk for severe ADAMTS-13 deficiency
- Total points= 6 or 7 – **high** risk for severe ADAMTS-13 deficiency

### Special Issues on aHUS Diagnosis

#### A) Role of the renal biopsy in aHUS

The main histopathological features of aHUS are: endothelial cell edema, subendothelial expansion due to edema or increase in matrix components and basement membrane detachment, accumulation of debris in the subendothelial space, and increased Von Willebrand factor expression, which attracts platelets and leads to the formation of microthrombi - which partially or completely occlude the lumen of vessels in the microvasculature. This occlusion leads to the mechanical destruction of erythrocytes by shear stress, which explains the intravascular anemia (intravascular hemolysis), platelet adhesion with thrombocytopenia, fragmented red blood cells (schistocytes) in the peripheral blood, and variable ischemia in the tissue.

**Figure 2:**
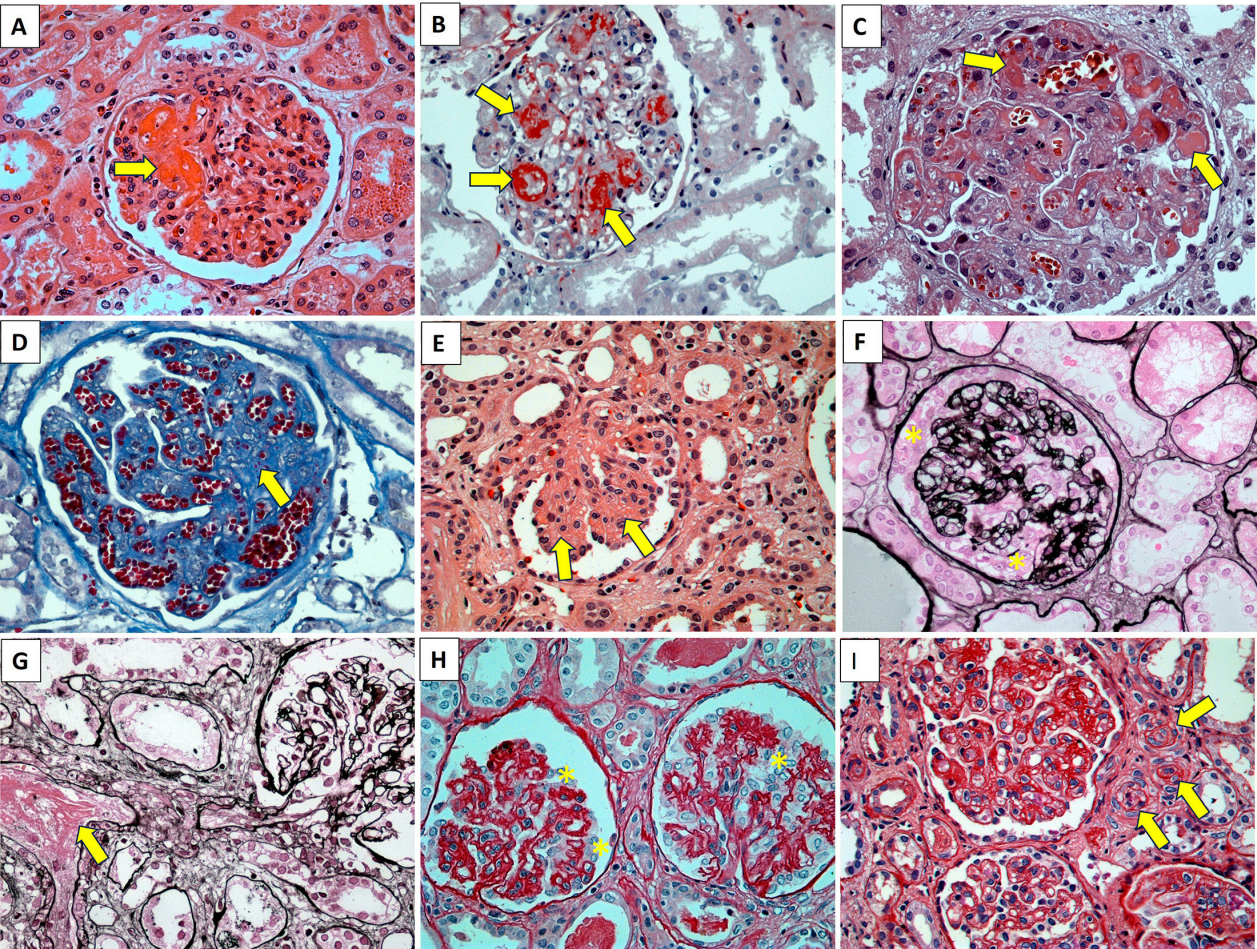
Acute, subacute, and chronic thrombotic microangiopathy in the glomeruli. Acute: A) H&E staining showing fibrin thrombus (arrows) in some glomerular tufts. There is diffuse endothelial edema and recruitment of leukocytes around the glomerular capillary loops. B) Masson’s trichrome staining revealing fibrin thrombus (arrows) obliterating the capillary loops together with endothelial edema and leukocyte permeation in the capillary loops. C) H&E showing duplicated capillary loops, endothelial edema, red blood cell fragmentation, foamy macrophages, and some fibrin thrombi obliterating the lumen of several glomerular tufts. D) Masson’s trichrome stain with congested capillary loops, duplication of the basement membrane, endothelial edema, and red blood cell fragmentation (schistocytes – arrow). Subacute: E) H&E showing a glomerulus with mesangial expansion with vacuolated matrix (mesangiolysis - arrows - resulting from thrombotic process and vascular repair). Chronic: F) Jones’ silver methenamine staining displaying a glomerulus with duplication of the glomerular basement membrane, endothelial edema, collapsed capillary loops with “podocyte hyperplasia” - asterisks. G) Jones’ silver methenamine staining identifying an interlobular arteriole with fibrin thrombus obstructing the vessel lumen. Downstream, the glomerulus with corrugated, ischemic/anemic capillary loops are visible. H) PAS staining revealing shrunken, ischemic, anemic glomeruli, with dilation of the urinary space and “podocyte hyperplasia” (resulting from upstream thrombotic vascular injury - asterisks). I) PAS displaying glomeruli with diffusely duplicated capillary loops, edematous endothelial cells (glomerular and vascular) with arterioles showing vascular lumen narrowing (arrows).

**Figure 3:**
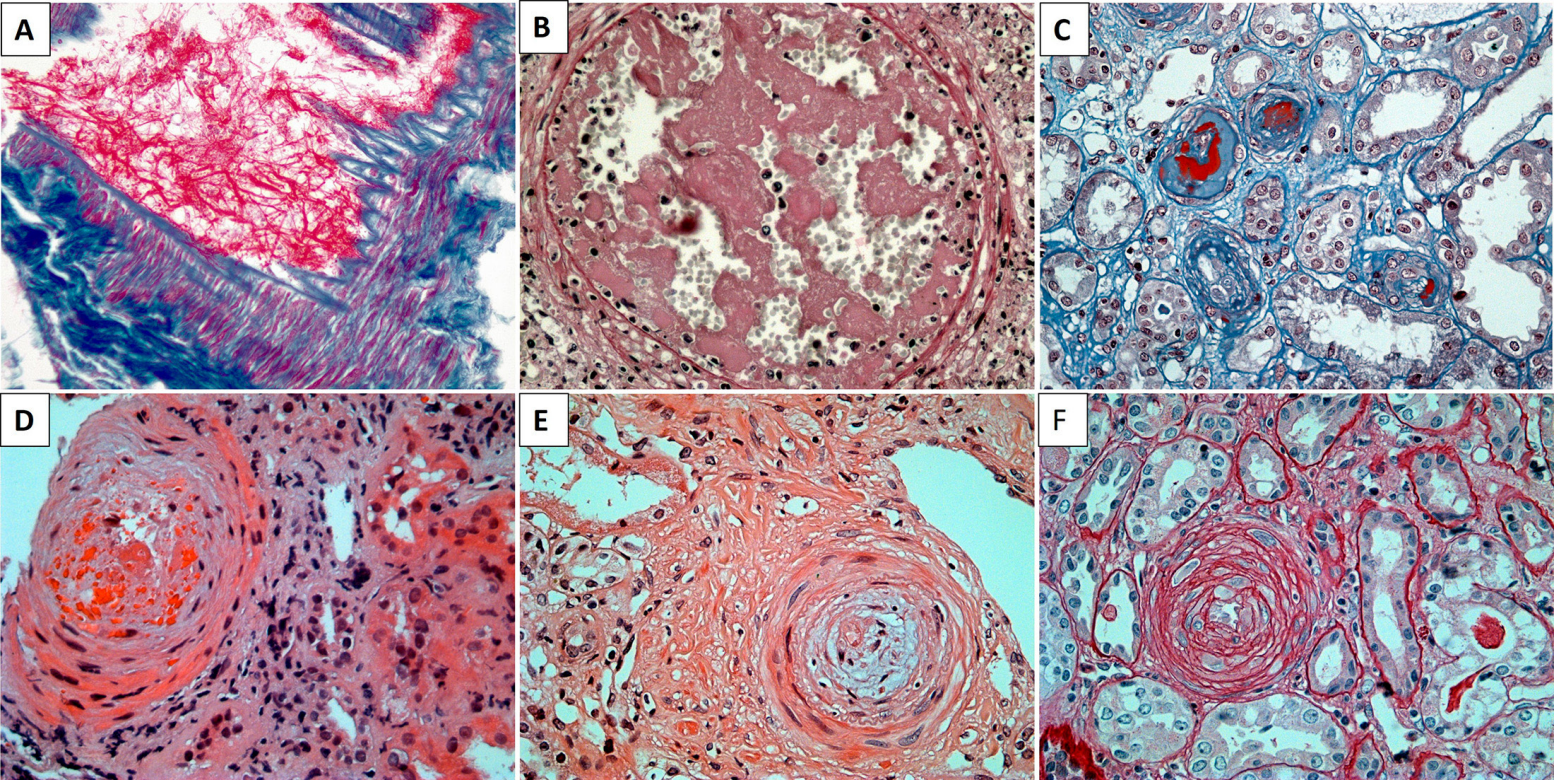
Acute, subacute, and chronic thrombotic vasculopathy. Acute A) Masson’s trichrome staining showing fibrin mesh adhered to the vascular endothelium and extending into the arterial vascular lumen. B) H&E staining showing the arterial vessel lumen with obliteration by a fibrin thrombus housing leukocytes, platelets, and whole and fragmented red blood cells. C) Masson’s trichrome staining displaying arteries with fibrin thrombi (in red) obliterating the vascular lumen. Subacute: D) H&E staining exhibiting an arterial vessel with diffusely edematous walls and fragmented red blood cells. E) H&E staining of arterial vessel with mucoid edema (pale/light blue) and obstruction of the vascular lumen. Chronic: F) PAS staining indicating an arterial vessel with onion skin lesion, characteristic of chronic endothelial/vascular damage.

**Figure 4:**
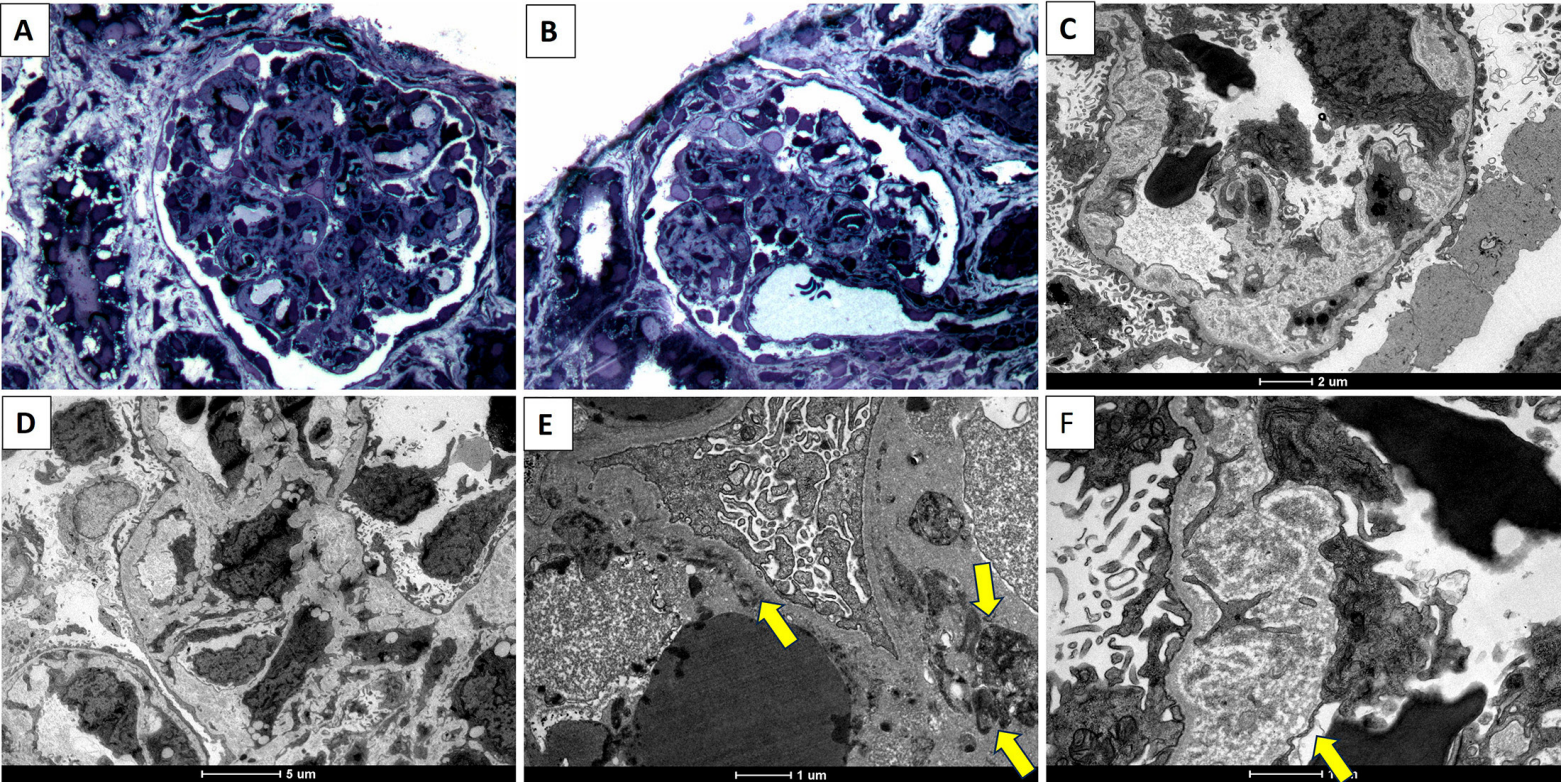
Thrombotic microangiopathic endothelial damage – ultrastructural analysis – A and B) Semi-thin section stained with Toluidine Blue showing glomeruli with edematous and duplicated capillary loops, endothelial edema, and narrowing of the vascular lumen. C to F Ultrathin sections analyzed by transmission electron microscopy contrasted with osmium tetroxide, uranyl acetate, and ruthenium red. C and D) Diffusely duplicated capillary loops, with expansion of the internal rarefied lamina by electronelucent material and hint of a newly formed basement membrane. E) Widening of the subendothelial space with deposition of fibrin tactoids (arrow). F) In detail, the expansion of lamina rara interna with lucent material and effacement of endothelial fenestrae (arrow) due to endothelial lesion.

**Figure 5:**
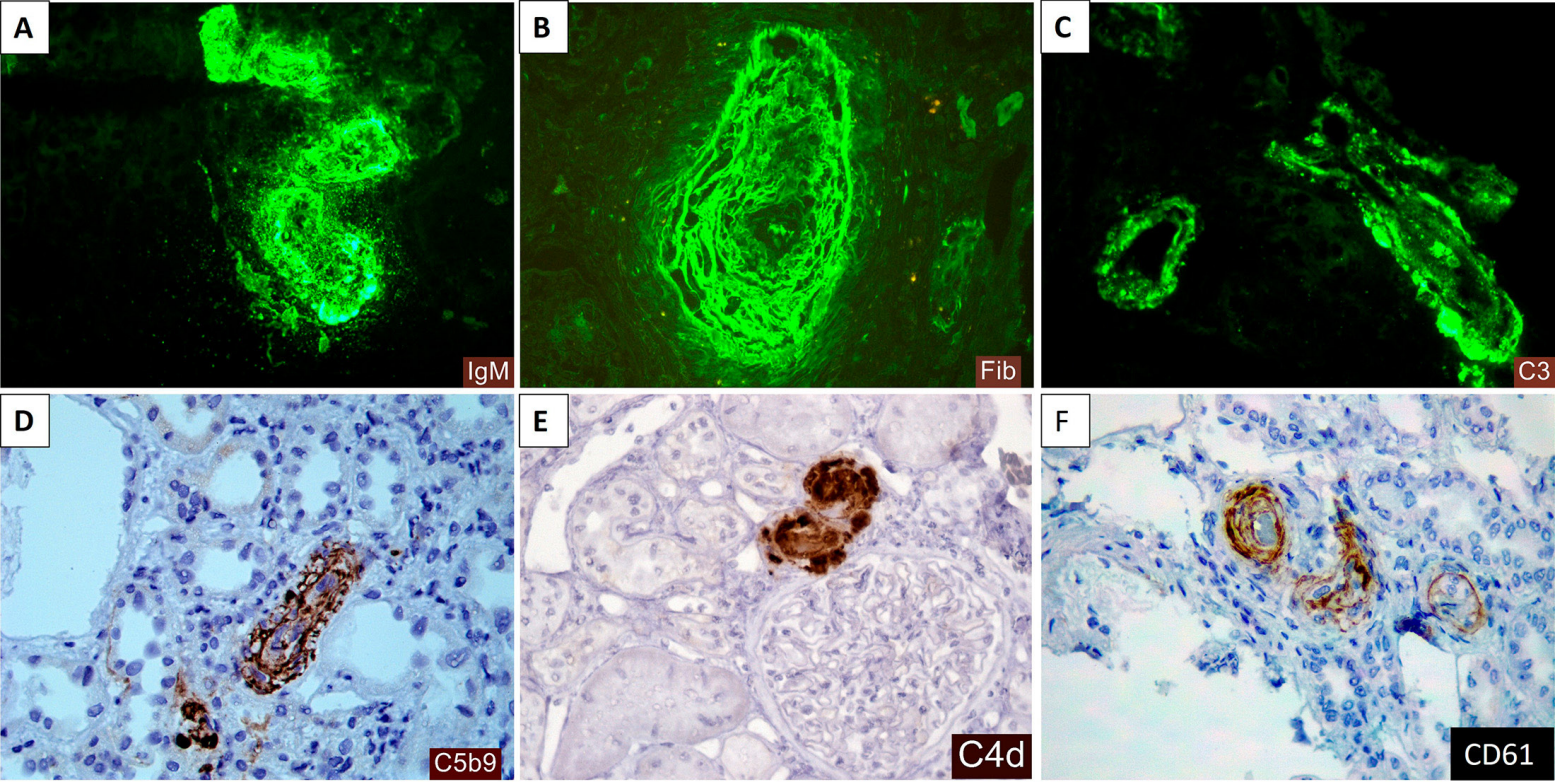
Thrombotic microangiopathy (immunophenotypic profile). A) Granular staining by immunofluorescence for IgM in the walls and lumen of arterial vessels with thrombotic vascular lesion (likely trapping within the thrombus mesh). B) Granular staining by immunofluorescence for fibrinogen in the walls of arterial vessels with thrombotic vascular lesion (resulting from endothelial damage and plasma/fibrin extravasation). C) Granular staining by immunofluorescence for C3 in vascular walls and vascular lumen (due to complement activation). D) Immunohistochemistry with deposition of membrane attack complex (C5b9) in the vascular lumen and wall. E) Immunohistochemistry with coarse granular staining in the walls of arterial vessels and vascular lumen (C4d complement fragment produced by classical and lectin pathway activation with high tissue stability). F) Immunohistochemistry for CD61 platelet aggregation marker with staining in walls and lumen.

Renal biopsy is not mandatory to diagnose TMA since there is a clinical correspondence with the triad MAHA, thrombocytopenia, and organ injury (particularly renal)^26^. However, it is recommended in special situations such as renal graft dysfunction in which the histological findings can discriminate between TMA and graft rejection, define the presence of underlying glomerulonephritis, and determine chronicity index to manage treatment expectations (grade 1B)^26^. Figures 2,3,4,5 show some examples of histological diagnostic criteria of TMA.

#### B) Role of genetic testing in aHUS

There is a known genetic basis for nearly two-thirds of aHUS cases, most of which are related to an inactivating mutation of the proteins that inhibit the alternative pathway: Factor H (*CFH*), Factor I (*CFI*), membrane cofactor protein (*MCP* or CD46), thrombomodulin (*THBD*), proteins related to Factor H 1 to 5 (*CFHR1-5*) or a gain-of-function mutation of activating factors of this complement pathway, *C3* or Factor B (*CFB*)^23^.

The formation of anti-CFH IgG antibodies has been found mostly in pediatric patients and is associated with genetic rearrangements (homozygous large deletions) in CFH-related proteins 1 and 3 (*CFHR1*-*CFHR3* deletion) in 87% of cases^19,27,28^.

In the Global Registry of aHUS^3^, approximately 40% of the 851 studied patients had no mutations or risk variants identified in complement genes. This may be due to alterations in other complement or coagulation genes, as demonstrated in an exome sequencing study conducted in 10 pediatric patients with aHUS^29^. In Brazil, 33.5% of patients who underwent genetic analysis were found to lack genetic variants^6,7^.

There is great variation among the groups and laboratories that carry out genetic analysis of aHUS, with the most common method being a next generation sequencing (NGS) panel containing genes from the alternative complement pathway (*CFH*, *CFI*, *CFB*, *C3*, *MCP*, *THBD*). Other laboratories also analyze coagulation genes (*PLG*, *DGKe*), large deletions or rearrangements of genes related to Factor H (*CFHR1* to *5*), and lectin pathway genes (*MASP2*). There is still no consensus regarding which genes should compose the ideal NGS panel.

In this context, findings from the aHUS Brazilian Registry largely coincide with those of the Global Registry, revealing a predominance of *CHF* variants across all age groups and an absence of CFI variants in pediatric patients^3^. However, a higher proportion of variants were identified in genes encoding Factor H-related proteins (*CFRH*) compared with other cohorts in Brazil^29,30^. The *CFHR1*-*CFHR3* large deletion was also detected in a high proportion of Brazilian patients. This finding suggests that Multiplex Ligation-Dependent Probe Amplification (MLPA), a gold standard for DNA copy number determination, should be performed in these patients, especially when no disease-related variant (grade 1B) has been detected by NGS^6,7^.

Patients often exhibit mutations in more than one gene or polymorphisms, potentially showing an additive effect of various genetic factors. Despite advancements, questions remain regarding genetic basis of aHUS, as the genotype-phenotype correlation may involve modifier genes, epigenetic events, and environmental factors. Some asymptomatic carriers have genetic alterations, while others with severe disease yield inconclusive genetic study results. While genetic analysis helps to understand the pathogenesis, negative findings do not rule out aHUS and the diagnosis relies on clinical markers^16^.

#### C) Overlap of aHUS-related genetic variants and other causes of TMA

aHUS-related genetic variants have already been described in patients with STEC-HUS^31^, pregnancy-associated TMA^32^, treatment-refractory autoimmune diseases^33^, hematopoietic cell transplantation^34^, and monoclonal gammopathy^35^.

Therefore, if TMA persists after treating the underlying disease or secondary TMA, concurrent aHUS^36^ or TTP^37^ should be explored, which affect therapeutic strategies and patient prognosis. Although a study of 110 patients with secondary TMA detected genetic findings like those of the general population of TMA patients^38^, other studies showed that many of the patients with secondary TMA refractory to treatment of the underlying disease responded to eculizumab, which was used only temporarily, with no TMA recurrence after withdrawal^39^.

## Management of TMA and aHUS

### Supportive Treatment

Supportive care follows AKI management principles: addressing volume/electrolyte balance, controlling hypertension, adjusting nephrotoxic drugs, initiating dialysis if indicated, and ensuring adequate nutrition. Severe anemia (Hb <7g/dL) requires blood transfusions, while platelet transfusions are reserved for active bleeding or surgical needs. Blood samples for direct Coombs test should be obtained before any transfusion. Additional supportive measures include dialysis, plasma exchange, and plasmapheresis/plasma infusion^40,41^ (grade 1B).

### Specific Treatment

Before the era of terminal complement inhibitors, aHUS management with supportive measures was considered ineffective, with 50% of patients requiring chronic dialysis and up to 25% of deaths occurring in the acute phase of the disease^42^. After the approval of the C5 inhibitor, eculizumab, by the FDA and the European Medicine Agency in 2011 among other agencies worldwide, including the *Agência Nacional de Vigilância Sanitária* (ANVISA, Brazilian Health Agency), eculizumab became the first-line therapy for this disease^41^. In the next section, we will discuss specific therapies for aHUS.

### Use of C5 Inhibitors – The Post-Eculizumab Era

All aHUS patients are eligible for C5 inhibitor therapy^18^, recommended as first line treatment (grade 1A). Initiation during the acute phase improves kidney function recovery^19^.

Eculizumab, a recombinant humanized monoclonal antibody targeting factor C5, blocks the complement system’s terminal portion, preventing C5b-9 formation, which damages endothelial cells^41^. Two-year prospective studies on eculizumab efficacy and safety demonstrated improvement of hemolysis, thrombocytopenia, and renal function. Patients with end-stage kidney disease (ESKD) treated with eculizumab showed fewer extrarenal manifestations and improved quality of life^19,41,43,44,45^.

Each Soliris^®^ vial (eculizumab’s commercial name) by Alexion Pharmaceuticals contains 300 mg in 30 mL solution for intravenous infusion over 35 minutes minimum^43,44,45,46^. Dosing and schedule are shown in Table 2.

**Table 2:** Eculizumab dosing and schedule for adult and pediatric patients with aHUS

Weight (kg)	Induction	Maintenance (per undetermined time)
≥40	900 mg per week for 4 weeks; 1200 mg in the 5th week.	1200 mg per week every 2 weeks.
≥30 to <40	600 mg per week for 2 weeks; 900 mg in the 3rd week.	900 mg per week every 2 weeks.
≥20 to <30	600 mg per week for 2 weeks; 600 mg in the 3rd week.	600 mg per week every 2 weeks.
≥10 to <20	600 mg per week for 2 weeks; 300 mg in the 3rd week.	300 mg per week every 2 weeks.
≥5 to <10	300 mg per week, for one week; 300 mg in the 2nd week.	300 mg per week every 3 weeks.

The side effects of this drug are associated with increased vulnerability to infections by encapsulated germs, especially *Neisseria meningitidis.* In addition to the use of prophylactic antibiotics, vaccination with tetravalent conjugate vaccine (MenACW135Y) and meningococcus B are recommended for all patients to protect against most meningococcal serotypes (at least 15 days before initiation of therapy). Other vaccines are also recommended, such as Pn13, Pn23, Hib, and influenza (grade 1A)^41^,[Bibr B42],^43^,^44^,^45^,^46^,^47^. We also recommend updating the vaccination schedule with booster doses. Although the manufacturer recommends antibiotics only for 15 days after vaccination, if vaccination was not possible before, we recommend using prophylactic antibiotics (against meningococcal disease) while the patient is under C5 inhibitor treatment (grade 1A).

Ravulizumab is a newly approved C5 inhibitor with a longer half-life that allows the maintenance dose to be extended to once every 4 weeks (for patients under 20 kg) or once every 8 weeks (for patients over 20 kg). The safety and efficacy of the medication in adults and children (over 10 kg) were confirmed in prospective trials^48,49^.

Ultomiris^®^ (ravulizumab’s commercial name) from Alexion Pharmaceuticals provides vials of 300 mg in 3 mL, 1100 mg in 11 mL, and 300 mg in 30 mL. In Brazil, only the 300 mg/3 mL option is available. Following dilution, the final concentration should be 50 mg/mL. Treatment comprises a loading dose followed by a maintenance phase two weeks later administered via intravenous infusion according to Table 3. Patients transitioning from eculizumab to ravulizumab should receive a loading dose of ravulizumab 2 weeks post-eculizumab’s final dose, followed by maintenance doses every 4 or 8 weeks based on weight, as previously outlined. When ravulizumab is used, longer intervals between infusions improve quality of life by minimizing punctures and displacements^48,49^.

**Table 3:** Ravulizumab dosing and schedule for adult and pediatric patients with aHUS

Weight (kg)	Loading dose (mg)	Maintenance* (per time indeterminate)	Minimum time infusion (loading dose/maintenance dose)
≥100	3000	3600 mg every 8 weeks	25/30 minutes
≥60 to <100	2700	3300 mg every 8 weeks	35/40 minutes
≥40 to <60	2400	3000 mg every 8 weeks	45/55 minutes
≥30 to <40	1200	2700 mg every 8 weeks	31/65 minutes
≥20 to <30	900	2100 mg every 8 weeks	35/75 minutes
≥10 to <20	600	600 mg every 4 weeks	45/45 minutes

Note – *First dose administered 2 weeks after a loading dose.

Ravulizumab was found to be more cost-effective than eculizumab, with further savings possible if a concentrated 100 mg/mL is used^50,51^.

**Figure 6:**
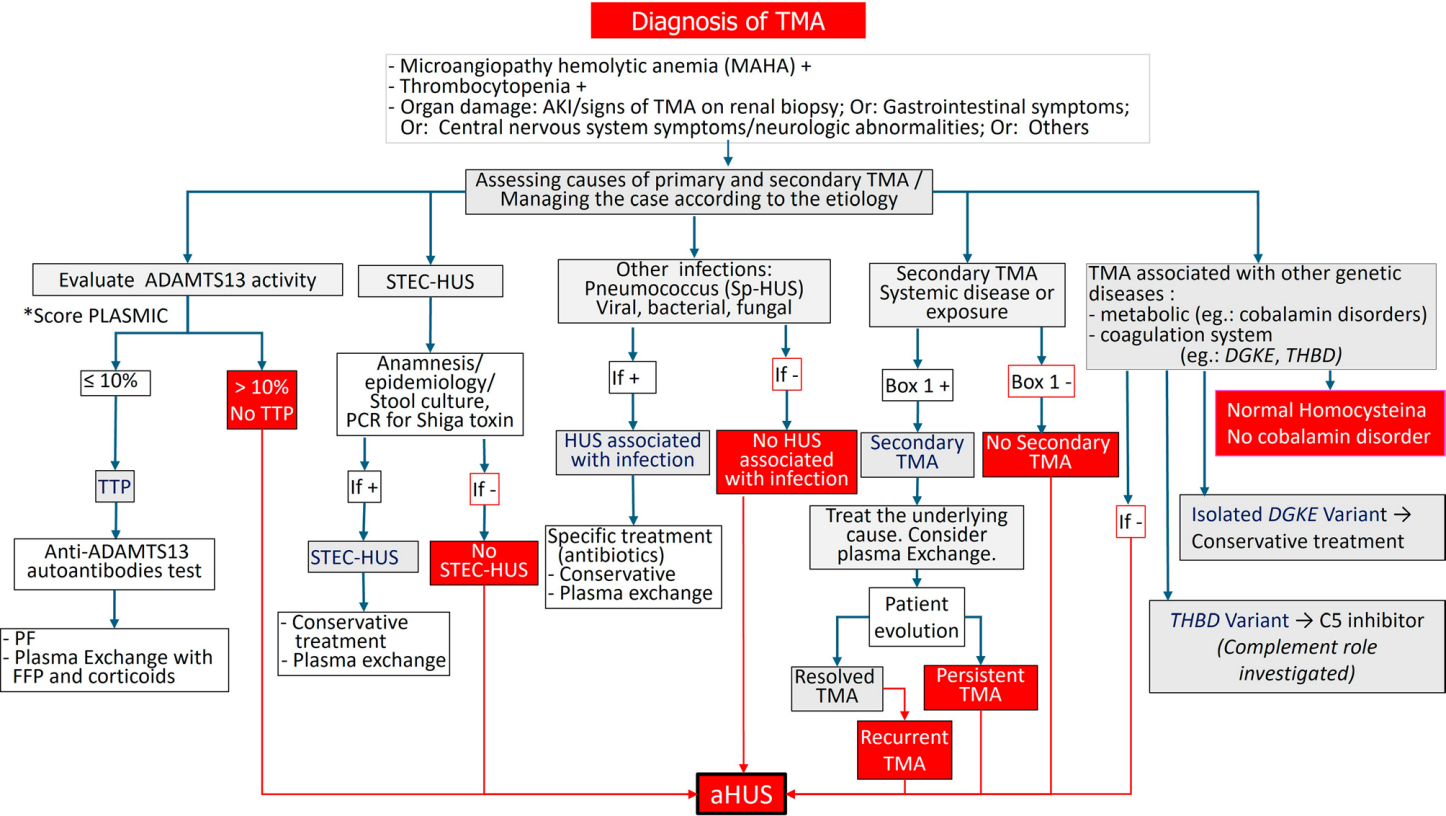
Diagnostic and therapeutic approach of TMA.

The recommended diagnostic and treatment criteria are shown in Figure 6 ^52^.

Abbreviations – TMA: thrombotic microangiopathy; TTP: thrombotic thrombocytopenic purpura; FFP: fresh frozen plasma; PCR: polymerase chain reaction; STEC: shiga toxin-producing Escherichia coli; aHUS: atypical hemolytic uremic syndrome; HUS: hemolytic uremic syndrome. Adapted from Claes *et al.,* 2018^51^.

### Monitoring Recommendation

Studies indicate that monitoring eculizumab complement blockade with CH50 levels can adjust infusion intervals for patients without disease recurrence. Jodele et al.^53^found that serum eculizumab levels correlated with CH50 in 365 paired samples from 18 bone marrow transplant patients, noting that a blood level above 99 **μ**g/mL suppressed CH50^53^.

Monitoring of complement blockade through CH50 inhibition for eculizumab is recommended (grade 1A). For ravulizumab, CH50’s reliability has not been proven; hence, clinical monitoring coupled with serum drug level measurement is advised (grade 1A).

### Dose Spacing

Ardissino et al.^54^ proposed that dose spacing should be monitored for patients maintaining CH50 lower than 30% without disease recurrence and/or organ damage. They suggested that a 0.75 mg/kg/day eculizumab dose maintains complement blockade for 4 weeks. Volokhina et al.^55^ evaluated 11 aHUS patients and their treatment spacing. With a 1200 mg maintenance at 4-5 week intervals, 80% had serum eculizumab levels higher than 50 **μ**g/mL. All patients with levels > 50 **μ**g/mL exhibited complete complement system blockage (CH50 lower than 10%)^55^.

Individualizing treatment with eculizumab serum levels between 50–100 **μ**g/mL and monitoring complement blockade via CH50 may be feasible^56^. Gatault et al.^57^ analyzed 7 patients who used eculizumab, and found that those under 90 kg had dosing intervals of 4 weeks and those under 70 kg had dosing intervals of up to 6 weeks^57^.

Dose spacing should be adjusted according to patient profile, comorbidities, treatment adherence, and available CH50 and/or serum eculizumab dosing (grade 1B). For ravulizumab, there are no studies that recommend dose spacing beyond the aHUS indications on the drug label.

In Brazilian clinical practice, due to the challenges of measuring serum anti-C5 drug level, it is recommended by this consensus that dose spacing should not be reviewed until 3 months of therapy onset, after hematological, renal, and systemic parameter improvement, with no sign of disease activity. Patient assessment should consider comorbidities, renal function, age, treatment adherence, commitment, and available genetic analysis (grade 2A). CH50 monitoring is essential, and without it, dose spacing is not recommended (grade 1B). Moreover, dose spacing is not recommended for kidney transplant patients (grade 1A). See Chart 4 for eculizumab dose spacing criteria.

**Chart 4:** Parameters to be verified for eculizumab dose spacing

Considerations involved in the management of eculizumab dose spacing	
Initial evaluation	Assess the patient’s clinical response to eculizumab, including improvement in hematological, renal, and systemic parameters.
Monitoring	Regularly monitor complement activity through CH50 levels and/or serum levels of eculizumab.
Timing of evaluation	Evaluate dose spacing only after at least 3 months from initiation of treatment, provided there is improvement in clinical parameters and absence of disease activity.
Patient profile assessment	Consider the patient’s profile, including comorbidities, renal function, age, adherence, and commitment to therapy.
Avoidance in kidney transplanted patients	The authors of this consensus do not recommend dose spacing for kidney transplant patients due to potential risk of disease recurrence.
Genetic analysis	If available, genetic test result can help predict the possibility of recurrence and its severity.
Follow-up	Continuously reassess the patient’s response to dose spacing.
Patient education	Educate the patient about the rationale behind dose spacing decisions to ensure their understanding and cooperation.

**Suggested management of eculizumab dose spacing:** CH50 must be measured the day before the subsequent dose. If CH50 is below 30%, the dose should be spaced by 3 weeks, with infusion normally administered on the third week. If the patient maintains normal test results (markers of TMA) and is asymptomatic, CH50 should be measured the day before the subsequent dose (the third week infusion). If CH50 is below 30%, spacing could be extended to the fourth week. Although some studies suggest spacing up to 6 weeks based on patient factors and weight, this consensus does not recommend intervals longer than 4 weeks (grade 2A).

For ravulizumab, the manufacturer recommends considering treatment interruption based on medical observation and patient profile after 6 months of treatment, in the absence of disease activity. A study on patients with paroxysmal nocturnal hemoglobinuria revealed efficacy in serum level assessment and longer infusion intervals of up to 10 weeks, reducing treatment costs by 37%^58^.

### Discontinuation of Therapy

The high cost of therapy, risk of potentially serious side effects (increased risk of meningococcal infection), and biweekly intravenous infusions in the maintenance phase, motivated studies on the discontinuation of eculizumab treatment^59,60^.

Many observational studies on eculizumab discontinuation emerged in the past decade. An Italian cohort of 16 aHUS patients who discontinued the drug reported 31.2% experiencing recurrence within 180 days, three of whom had a *CFH* variant^29,61^. In a French cohort, 31% of 38 patients relapsed within 22 months after therapy cessation, with *CFH* mutation correlating with more severe manifestation and early recurrence^30^.

A Dutch cohort study on restricted eculizumab use in 20 aHUS patients observed a 25% recurrence rate over 1460 days^62^. The researchers developed a mathematical tool for individualized eculizumab dosing and spacing during maintenance, guided by therapeutic drug monitoring. With this approach, equivalent therapeutic outcomes and cost-effectiveness were achieved, reducing therapy costs by up to 13%^62,63^. Additionally, early eculizumab initiation (within 3 months) in aHUS patients with native kidney involvement yielded a 19% recurrence rate, with cost savings of up to 30%^64^.

The first prospective cohort study was published by Fakhouri et al*.* ^65^ in 2021 and involved 55 patients from different French centers. It had a recurrence rate of 23% and only 3 patients had kidney transplant^65^.

In a systematic review, Macia et al.^66^ analyzed published cases, unpublished data, clinical studies, and data from the Global aHUS Registry. Recurrence episodes were found in 4 (66.6%) of the 6 patients in unpublished case reports and 16 (30.7%) of 52 patients in published case reports. In clinical studies, recurrence occurred in 12 (19.6%) of 61 patients, 5 (41.6%) of whom had a *CFH* mutation. Finally, the global registry showed 12 (15.7%) recurrences in 76 patients who discontinued eculizumab therapy^66^.

A Brazilian cohort of aHUS patients who had unplanned eculizumab discontinuation found a cumulative recurrence incidence of 58% in almost 400 days of follow-up. Patients with native kidney, transplant recipients, and dialysis patients were included^67,68^.

While there are no definitive guidelines on discontinuing therapy and timing in the literature, this consensus recommends planned discontinuation if genetic testing, complement system component evaluation (e.g., CH50 and C5b9), or therapeutic drug level are available. Furthermore, the immediate availability of the drug for reintroduction in the event of a relapse is mandatory^62,63,64,65,68^ (grade 1C).

We recommend shared decision making between the medical team and the patient regarding eculizumab discontinuation (grade 1A). Safety data on discontinuation remains inconclusive for determining patient eligibility and timing.

Whenever possible, we recommend laboratory evaluation of drug therapeutic levels and components of the complement system, at least serum CH50 dosage (grade 1A). In addition, we recommend immediate access to drugs to treat patients with recurrence (grade 1A).

## Future Perspectives: New Complement Inhibitors

### Pegcetacoplan

Pegcetacoplan is a new complement inhibitor approved by the FDA in 2021 for paroxysmal nocturnal hemoglobinuria. This drug binds to the C3 component of the complement system, preventing its cleavage and activation. The recommended dose is based on weight, and for adults is subcutaneous administration of 1080 mg twice a week. It is being studied for C3 glomerulopathy, macular degeneration, and autoimmune hemolytic anemia, with good results^69^. There are still no studies for aHUS, but as it is a proximal complement blocker, it is believed to be beneficial^69^.

### Iptacopan

Iptacopan is a potent CFB inhibitor that acts on the complement alternative pathway^70^. There are some studies evaluating this drug in complement dysregulation disease such as C3 glomerulopathy^71^, demonstrating improvement in proteinuria^72^.

Also, a phase II clinical trial is currently underway to evaluate Iptacopan in patients with aHUS, but no results are yet available. However, this could be another possibility for this treatment.

### Crovalimab

Crovalimab (RO7112689 or SKY59; marketed by Chugai Pharmaceutical) is a novel anti-C5 sequential monoclonal antibody recycling technology (SMART) antibody that combines isoelectric point, neonatal Fc receptor, and pH-dependent affinity engineering^73^. This results in efficient C5 binding, enhanced uptake of C5-bound crovalimab by endothelial cells, disposal of C5 in the endosome, and efficient neonatal Fc receptor-mediated recycling of crovalimab. Furthermore, crovalimab is highly soluble, allowing for small injection volumes^73^. Crovalimab binds to the C5 β-chain and prevents cleavage of the wild-type and SNP C5 by the C5 convertase. Two clinical trials are under way for aHUS patients (NCT04958265 and NCT04861259), and are recruiting pediatric, adolescent, and adult patients. This medication has great potential for a good response in aHUS patients^73^.

### Eculizumab Biosimilars (Elizaria)

Elizaria, developed by IBC Generium, Russia, is the world’s first registered biosimilar of eculizumab (Soliris^®^, marketed by Alexion Pharmaceuticals)^74^. A multitude of analyses revealed that the amino acid sequence is identical and higher-order structures, post-translational modifications, purity, and product variants are highly similar between Elizaria^®^ DP and Eculizumab RP, except for minor differences in the relative abundance of the charge variants and glycan moieties, which are not considered clinically significant^74^. However, due to the limited experience with this drug worldwide, this consensus recommends the use of reference anti-C5 inhibitors such as eculizumab or ravulizumab instead of biosimilars.

### Narsoplimab

Narsoplimab is a humanized anti-MASP2 monoclonal antibody. MASP2 is a serine protease associated with the mannose pathway that binds to the complement lectin pathway. It is believed that the hyperactivation of MASP2 stimulates the lectin pathway, mainly in autoimmune diseases, TMA associated with bone marrow transplantation (BMT), and infections^75^. This medication is indicated for TMA related to BMT, following evidence from a phase II study^76^. There is no relevant evidence for use in patients with aHUS.

## Special Situations

### Pediatric

Establishing the diagnosis and etiology of TMA in children is important for immediate disease management. Although there is an overlap of TMA etiologies in adults and children, some of the diseases are more common in children, while others only occur children^77^.

The main cause of TMA in children is STEC-HUS, followed by aHUS and Sp-HUS^78^. Especially in children under 2 years of age, there are rare conditions such as congenital TTP (caused by variants in the gene *ADAMTS13)*, cobalamin metabolic disturbances (caused by variants in the gene *MMAHC,* C cobalamin defects or *MTA,* G cobalamin defects^79^), and coagulation disorders that must be ruled out before the diagnosis of aHUS^77^.

In neonates, perinatal asphyxia is a critical differential diagnosis that can confirm TMA. Perinatal abnormalities (due to fetal, maternal, or placental reasons) can impair fetal or neonatal gas exchange, triggering TMA (MAHA, thrombocytopenia, and several organ injuries, mainly renal)^80^. Delayed treatment can result in severe organ compromise, including cardiac, hepatic, and renal insufficiency, vascular lesions, and encephalopathy^80^.

Signs of disseminated intravascular coagulopathy (DIC) are critical in asphyxiated newborns^80^, indicating consumption coagulopathy due to ischemia/hypoxia^81^. Perinatal asphyxia markers include low Apgar score, metabolic acidosis (detected early in umbilical cord blood) and multiple organ failure^82,83^ (grade 1B). However, the clinical overlap between neonatal aHUS and perinatal asphyxia complicates diagnosis. aHUS can also lead to asphyxia and cerebral damage in newborns, making identification of the primary event difficult^80,83^. Maternal and gestational history, placental appearance, birth conditions, Apgar score, and early metabolic acidosis are crucial in clinical practice. Low plasma C3 levels suggest hyperactivation of the alternative complement pathway. aHUS is the main diagnosis in cases of TMA recurrence^81^, more severe neurological involvement^80^, and an accelerated and not-consumptive disease^83^.

Clinicians should be vigilant for TMA development in asphyxiated newborns, initiating appropriate treatment to reverse TMA. However, persistent TMA warrants consideration of neonatal aHUS (grade 1B).

### Clinical Manifestations and Particularities of aHUS Therapy in Pediatrics

Children exhibit significantly lower levels of hemoglobin and platelets and higher LDH compared to adults^7,84^, indicating a potentially more severe hemolytic effect in childhood. Moreover, children have a higher mortality rate than adults^7,83^.

The anti-C5 monoclonal antibody (mAb) eculizumab is the first line therapy for aHUS in children^46,79,82,85,86^, and it has been demonstrated to be safe and effective by many clinical trials, cohort studies, and case reports. Especially in children, eculizumab has promoted TMA remission and it is frequently associated with complete recovery of the renal function^46,85,86^.

If the anti-C5 mAb is not immediately available at the emergency department, plasma therapy should be initiated, including plasmapheresis or plasma infusion (grade 1B); the choice depends on the appropriate conditions of the service, professional experience, clinical status, and child size. Although plasma therapy has not been shown to be effective in maintaining long-term remission and promoting renal function recovery, it may transiently improve TMA by providing complement regulatory proteins and, in the case of plasmapheresis, it is possible to remove CFH antibodies^87^.

However, it is important to emphasize the morbidity associated with this procedure, especially in children, linked to venous central catheterization complications and hypervolemia^88^.

Hydroxycobalamin can be administered in an emergency, while test results are not available. Although cobalamin disturbances leading to TMA are rare, they are treatable and there is no severe adverse event^83^.

Currently, other anti-C5 blockers have been studied in children. Ravulizumab is now approved and there are pediatric clinical trials showing its efficacy and safety^89^. Other options are now under investigation, with better posology and the possibility of subcutaneous (crovalimab) or oral (iptacoplan) administration.

### Pregnancy

Pregnancy-associated TMA is a rare disorder with an estimated incidence of approximately 1 in 25,000 pregnancies and it is associated with significant perinatal and maternal morbidity and mortality^90^.

Pregnancy and postpartum have long been recognized as high-risk conditions for TMA. There are three main differential diagnoses for pregnancy-associated TMA: (1) Pre-eclampsia/hemolysis, elevated liver function tests, low platelet syndrome (PE/ HELLP); (2) TTP; and (3) aHUS. Pregnancy is a known trigger for TTP and aHUS, and the presence of these disorders increases the risk of PE/HELLP syndrome.

For TMA markers, some experts propose a lower platelet count threshold for clinical diagnosis, considering that in normal pregnancy platelets decrease. Approximately 10% of uncomplicated pregnancies have platelet counts below 150,000/mm^3^ at delivery. Hence, a threshold of 100,000/mm^3^ appears to be appropriate for diagnosing pregnancy-associated TMA^91^. Other parameters such as anemia, elevated LDH, reduced haptoglobin, presence of schistocytes, and organ damage align with recommendations for other TMA forms.

AKI is frequently found in most types of pregnancy-associated TMA, except TTP. Although there is no universally accepted definition of AKI during pregnancy, the various definitions available refer to the KDIGO guidelines^19^. Other publications are based on a serum creatinine above 0.90 mmol/L and/ or a 0.25% increase from baseline^92^.

### aHUS in Pregnancy

Pregnancy is a condition of increased activity of all pathways of the complement system, including classical, lectin, and alternative pathways. The aim is to clear the maternal circulation of immune complexes and, on the other hand, of regulatory proteins for complement control (mainly MCP and CD59). Also, studies have identified variants in complement system genes in more than 50% of pregnancy-associated TMA^93^.

aHUS, the rarest form of TMA in pregnancy, often arises in late third trimester or postpartum. Cases outside these periods complicate differential diagnosis with PE/HELLP^91,94^. Renal impairment is common, while platelet count is usually not critically reduced, and neurological involvement, unlike TTP, is infrequent^91,94,95^.

Currently, the recommended treatment is a C5 inhibitor (grade 1B). Without this treatment, renal outcomes are dismal, with 76% of patients progressing to end-stage kidney disease (ESKD) despite receiving plasmapheresis^93,94^. Another study showed a 50% risk of ESKD in pregnant women with aHUS, regardless of whether they underwent plasmapheresis or not^94^.

Despite the high cost of the medication, it generally does not exceed the cost of intensive care treatment, plasmapheresis, hemodialysis, probable kidney failure, and transplantation^96^. Anti-C5 mAb can cross the placenta, but data limited to the number of pregnancies exposed to eculizumab (fewer than 300 pregnancy outcomes) indicate that there is no increased risk of fetal malformation or fetal-neonatal toxicity^96,97^. No controlled clinical study has been carried out to evaluate the efficacy of anti-C5 in pregnancy-associated aHUS. Despite this, more than 35 cases have been reported in the literature in which eculizumab was administered during or after pregnancy, with approximately 90% showing hematological response and remission of kidney disease^97^.

Treatment duration is uncertain, and discontinuation of anti-C5 treatment should be personalized. Complement gene variants increase the risk of recurrence. Terminal complement blockade must be monitored since pregnancy may require higher dose/frequency due to volume changes, increased C5 synthesis, or proteinuria. Despite eculizumab, prior aHUS history elevates risk of recurrence in subsequent pregnancies, requiring vigilant monitoring^93^.

According to the label, ravulizumab is considered Category C during pregnancy (pregnant women should not use this medication without medical advice). There are no clinical data on exposure in pregnancy. However, recent studies report the effectiveness and safety of ravulizumab in postpartum aHUS^98^.

## Transplant

### Pre-Transplant Investigation

Stage 5 CKD patients with unknown cause, post- pregnancy cases, lupus nephritis, TMA histology, and malignant hypertension should be considered potential aHUS cases. Pre-transplant assessment should include blood count with schistocytes, LDH, Coombs test, haptoglobin, autoantibodies and complement levels (C3 and C4)^99^.

If aHUS is likely and hemolysis evident (active aHUS), a 6-month course of anti-C5 mAb before transplantation should be considered to evaluate potential kidney function recovery^99^ (grade 1C).

### Genetic Analysis in Transplantation

Genetic analysis of all potentially linked genes helps medical teams and patients in devising strategies to prevent post-transplant aHUS recurrence^100^ (grade 1C).

The risk of recurrence of aHUS in kidney graft correlates with genetic variant type. Kidney transplantation in aHUS and ESKD patients is intricate, with relapse rates of 50–80%^41,42^ resulting in graft loss in up to 91.6% of cases^42,43,98^. Transplant recipients are at TMA risk from factors damaging the endothelium, including immunosuppressive drugs (calcineurin inhibitors and mTOR inhibitors), ischemia-reperfusion injury, rejection, and post-transplant infections^101^.

After the genetic tests, patients must be stratified into recurrence risk groups (grade 1A), and the best prophylactic regimen should be addressed before the surgery. High-risk patients are those with previous transplant recurrence, disease-related variants in *CFH*, or gain-of-function variants in *CFB* or *C3*. Moderate-risk patients have anti-factor H antibodies, *CFI* variants, uncertain significance variants, or *CFH* polymorphisms. Low-risk patients have *MCP* mutations, persistently negative factor H antibodies, or no mutations/polymorphisms^101^ (Chart 5)^99^ and one can observe transplant outcomes of these patients using eculizumab, if needed.

**Chart 5:** Pre-transplant aHUS investigation algorithm and recurrence risk

Pre-transplant aHUS Investigation algorithm		
Suspected patients	Additional tests	Consider
**Malignant hypertension postpartum biopsy showing TMA lupus nephritis**	HMG w/schistocytes DHL Coombs test Haptoglobin C3/C4 Autoantibodies antiphospholipids **Extra renal history** Cardiomyopathy Cerebrovascular Thrombosis	Genetic analysis
Probable diagnosis		
**Probably active aHUS** (evidence of hemolysis)	Consider eculizumab for 6 months	**No answer:** Transplant list (Assess recurrence risk)
**Probably aHUS**(no evidence of hemolysis)	Transplant list (Assess recurrence risk)	
Risk of post-transplant recurrence1		
High risk	Moderate risk	Low risk
– Recurrence after transplant – Inactivating variant in *CFH* – Gain of function variant in *CFB* or *C3*	– Anti-factor H antibody – Variant in CFI gene – Variants of uncertain significance (VUS) – *CFH* polymorphisms	– *MCP* variants – Negative factor H antibody – No mutation or polymorphisms
**Eculizumab pre-transplantation**	**Eculizumab pre-transplantation**	**Monitoring transplantation without eculizumab**
In all cases of suspected aHUS		
**• Avoid using expanded criteria donor** **• Avoid transplantation in the presence of anti-donor antibodies** **• Introduce cytomegalovirus prophylaxis** **• Avoid using mTOR inhibitors in combination with calcineurin inhibitors** **• Avoid high doses of calcineurin inhibitors**		

Adapted from Zuber *et al*. 2013^99^.

Using living related donors is not recommended for aHUS patients due to potential donor variant risks after nephrectomy (grade 1B). If considering a related donor, genetic analysis should ensure no complement gene variants. Discussing post-nephrectomy aHUS risks with the potential donor is crucial (grade 1B).

Additionally, it is recommended to avoid mTOR inhibitors with calcineurin inhibitors, high calcineurin inhibitors doses, anti-donor antibodies in transplantation, expanded criteria donors, and prolonged cold ischemia times (grade 1B). These strategies aim to mitigate graft endothelial stress, reduce ischemia-reperfusion injury, and potentially decrease activation of the alternative complement pathway.

### Diagnosis of Post-Transplant aHUS

The diagnosis of post-transplant aHUS is similar to that in the general context. However, some special secondary causes should be ruled out, such as those induced by calcineurin/mTOR inhibitors as well as antibody-mediated rejection and autoimmune and viral diseases (Chart 6)^102^.

**Chart 6:** Steps to diagnose atypical hemolytic uremic syndrome (aHUS) after transplantation

Microangiopathic hemolytic anemia (MAHA)	
**ADAMSTS13**	>5% rules out severe disability
**Discontinuation or dose reduction of calcineurin inhibitors and/or mTOR Inhibitors**	Persistent microangiopathy suggests aHUS
**Exclusion of viral infections(HIV; HTLV I/II; hepatitis B; hepatitis C; cytomegalovirus; Epstein-Barr)**	
**Exclusion of bacterial infections(blood culture, urine culture, stool culture)**	
**Exclusion of autoimmune diseases(FAN, antiDNAn, ANCAc, ANCAp, rheumatoid factor)**	
**Histology suggestive of microangiopathy**	
**Exclusion of antibody-mediated rejection**	Negative C4d test on biopsy and absence of donor antibody
**Diagnosis of aHUS**	
**Complement study with genetic analysis**	

Adapted from Andrade *et al*., 2017^102^.

Daily laboratory tests are advised until normal hematological parameters are obtained and renal function improves. Hemolytic anemia tests include blood count, platelet count, peripheral blood smear (for schistocytes), LDH, and haptoglobin. Renal function monitoring involves serum creatinine and urinary protein/creatinine ratio measurements (grade 1B).

### Treatment Recommendations After Transplantation

Eculizumab is effective in post-kidney transplantation cases of aHUS^101,103–105^. Ravulizumab also is also effective and safe in transplant patients, as per case reports. The recommended dose of these drugs for kidney transplant patients is the same as that for other patients. Immunosuppression with calcineurin inhibitors is advised with careful monitoring to prevent overexposure (grade 1B), while mTOR inhibitors should be avoided in aHUS patients undergoing kidney transplantation (grade 2B). Long-term belatacept can be used to avoid calcineurin inhibitors, but this drug in not regularly available in Brazil.

## Conclusion

The COMDORA-SBN expert group provides recommendations for the diagnosis and treatment of aHUS in the Brazilian population. These guidelines aim to improve, rather than restrict, current clinical practices. This consensus will be regularly updated with new information and data as needed.
