# Supplementary material for: Recommendations for diagnosis and treatment of Atypical Hemolytic Uremic Syndrome (aHUS): an expert consensus statement from the Rare Diseases Committee of the Brazilian Society of Nephrology (COMDORA-SBN)
Source: J Bras Nefrol. 2025 Feb 7;47(2):e20240087. doi: 10.1590/2175-8239-JBN-2024-0087en (PMC11804885; doi:10.1590/2175-8239-JBN-2024-0087en)
Supplement: Translation

# Translation: Recomendações para diagnóstico e tratamento da Síndrome Hemolítico-Urêmica Atípica (SHUa): uma declaração de consenso de especialistas do Comitê de Doenças Raras da Sociedade Brasileira de Nefrologia (COMDORA-SBN)

**DOI:** 10.1590/2175-8239-JBN-2024-0087pt

**Authors:** Maria Helena Vaisbich, Luis Gustavo Modelli de Andrade, Maria Izabel Neves de Holanda Barbosa, Maria Cristina Ribeiro de Castro, Silvana Maria Carvalho Miranda, Carlos Eduardo Poli-de-Figueiredo, Stanley de Almeida Araujo, Miguel Ernandes, Maria Goretti Moreira Guimarães Penido, Roberta Mendes Lima Sobral, Oreste Ferra, Precil Diego Miranda de Menezes Neves, Cassiano Augusto Braga da Silva, Fellype Carvalho Barreto, Igor Gouveia Pietrobom, Lilian Monteiro Pereira Palma

**Affiliations:** 1 Universidade Federal de São Paulo, Instituto da Criança, Hospital das Clínicas - HCFMUSP, São Paulo, SP, Brasil.; 2 Universidade Estadual Paulista, Departamento de Medicina Interna, São Paulo, SP, Brasil.; 3 Hospital Federal de Bonsucesso, Serviço de Nefrologia e Transplante Renal, Rio de Janeiro, RJ, Brasil.; 4 Universidade de São Paulo, Unidade de Transplante Renal, São Paulo, SP, Brasil.; 5 Hospital Santa Casa de Belo Horizonte, Belo Horizonte, MG, Brasil.; 6 Pontifícia Universidade Católica do Rio Grande do Sul, Porto Alegre, RS, Brasil.; 7 Universidade Federal de Minas Gerais, Instituto de Nefropatologia, Centro de Microscopia Eletrônica, Belo Horizonte, MG, Brasil.; 8 Hospital Beneficiência Portuguesa de São Paulo, São Paulo, SP, Brasil.; 9 Universidade Federal de Minas Gerais, Nefrologia Pediátrica, Centro de Nefrologia da Santa Casa de Belo Horizonte, Belo Horizonte, MG, Brasil.; 10 Universidade Federal da Bahia, Hospital Universitário Prof. Edgard Santos, Unidade do Aparelho Urinário, Salvador, BA, Brasil.; 11 Universidade Federal de Mato Grosso do Sul, Hospital Universitário Maria Aparecida Pedrossian, Campo Grande, MS, Brasil.; 12 Universidade de São Paulo, Departamento de Nefrologia, São Paulo, SP, Brasil.; 13 Hospital Alemão Oswaldo Cruz, Centro de Diálise e Nefrologia, São Paulo, SP, Brasil.; 14 Clínica Senhor do Bonfim, Departamento de Nefrologia, Feira de Santana, BA, Brasil.; 15 Universidade Federal do Paraná, Curitiba, PR, Brasil.; 16 Universidade Federal de São Paulo, São Paulo, SP, Brasil.; 17 Universidade Estadual de Campinas, Nefrologia Pediátrica, Campinas, SP, Brasil.

**Keywords:** Diretrizes, Diagnóstico, Tratamento, Síndrome Hemolítico-Urêmica Atípica, Microangiopatia Trombótica

## Abstract

A síndrome hemolítico-urêmica atípica (SHUa) é uma causa rara de microangiopatia trombótica (MAT) causada pela desregulação da via alternativa do complemento. O diagnóstico de MAT é feito clinicamente pela tríade: anemia hemolítica microangiopática, trombocitopenia e lesão de órgãos (principalmente injúria renal aguda). A heterogeneidade das manifestações clínicas e a ausência de um teste diagnóstico padrão-ouro tornam o diagnóstico preciso da SHUa um processo desafiador, podendo ter impacto no manejo do paciente. Até uma década atrás, não havia tratamento específico para a SHUa e os pacientes eram submetidos à terapia plasmática (troca de plasma e/ou infusão de plasma) e/ou transplante de fígado, procedimentos que não estão isentos de complicações graves e que não abordam a fisiopatologia subjacente da doença. Desde 2011, um anticorpo monoclonal anti- complemento C5 foi aprovado pela *Food and Drug Administration* (FDA) para pacientes com SHUa, dando início a uma nova era no tratamento. Ensaios clínicos sobre novos inibidores do complemento também podem aumentar o portfólio de tratamentos no futuro. A população brasileira é miscigenada, com um perfil genético e clínico único. Este consenso tem como objetivo oferecer recomendações para o diagnóstico e tratamento de pacientes com SHUa nesta população, com base na experiência de especialistas, dados do Registro Brasileiro de SHUa e revisão da literatura. O sistema GRADE foi utilizado para classificar a qualidade das evidências.

## Introdução

A síndrome hemolítico-urêmica atípica (SHUa) é uma causa ultrarrara de microangiopatia trombótica (MAT), caracterizada por anemia hemolítica não imune, trombocitopenia e manifestações sistêmicas, incluindo envolvimento renal, frequentemente manifestado como injúria renal aguda (IRA). Anormalidades nas proteínas reguladoras da via alternativa do complemento levam à formação excessiva do complexo de ataque à membrana (C5b-9), causando danos às células endoteliais e formação de microtrombos em todo o corpo^1^. Variantes relacionadas à doença em genes reguladores do complemento ou a presença de autoanticorpos contra o fator H do complemento (CFH) são encontradas em 60–70% dos pacientes^2^. Embora haja uma tendência na literatura de utilizar o termo SHU mediada por complemento, optamos por aderir ao termo SHUa neste consenso, conforme definido em estudos pivotais sobre inibidores do complemento e pela *Food and Drug Administration* (FDA).

A epidemiologia da SHUa é influenciada pelo contexto genético e pelas características da população^3,4^. Os dados globais são limitados devido à raridade da SHUa. Uma revisão sistemática de 2020 forneceu as primeiras percepções epidemiológicas consistentes^5^. Dados da Noruega, França, Itália e Austrália estimaram a prevalência e a incidência de SHUa. A prevalência entre indivíduos com 20 anos ou menos variou de 2,2 a 9,4 por milhão, com uma prevalência geral de 4,9 por milhão^5^. As taxas de incidência anual para indivíduos com mais de 20 anos variaram de 0,26 a 0,75 por milhão e, para todas as idades, de 0,23 a 1,9 por milhão^5^.

A ascendência genética diversificada da população brasileira e sua taxa de miscigenação elevada tornam sua população ideal para ampliar o espectro genético da SHUa^6,7^. O Registro Brasileiro de SHUa, coordenado pelo Comitê de Doenças Raras da Sociedade Brasileira de Nefrologia (COMDORA-SBN), revelou um perfil de doença exclusivo^7^. Afetando predominantemente mulheres e adultos jovens, observou-se uma alta taxa de envolvimento renal. Pacientes pediátricos apresentaram níveis mais baixos de hemoglobina e plaquetas na apresentação, além de níveis mais elevados de LDH em comparação com adultos. Variantes genéticas comuns, particularmente no gene *CFH* e uma ampla deleção *CFHR1-3*, foram encontradas em todas as faixas etárias^7^, o que tem implicações para a escolha dos métodos de teste genético.

As manifestações clínicas dependem da gravidade da isquemia nos órgãos afetados^8^. Associado à condição hematológica, o envolvimento renal é frequentemente observado, manifestando-se como injúria renal aguda, edema, oligoanúria, proteinúria, hematúria e hipertensão arterial sistêmica. Além disso, pode haver envolvimento do sistema nervoso central (confusão mental, letargia, convulsões, coma), distúrbios do trato gastrointestinal (diarreia, distúrbios hepáticos, pancreatite), envolvimento pulmonar levando à hemorragia alveolar, complicações oculares (amaurose), isquemia cutânea (que pode levar à necrose das extremidades) e envolvimento cardíaco^9,10^.

É importante ressaltar que, em alguns casos, pode ocorrer uma apresentação subaguda com comprometimento renal e hipertensão arterial com sinais de MAT na biópsia renal, mas sem sinais sistêmicos de hemólise e trombocitopenia. Portanto, o diagnóstico diferencial de MAT deve ser considerado em qualquer paciente que apresente injúria renal e hemólise de pequeno grau (grau 1B)^11^.

A SHUa é bastante heterogênea em sua manifestação clínica, resultando em dificuldades tanto no diagnóstico quanto no tratamento^1^. Para abordar essas questões, um grupo de especialistas apresenta o primeiro documento de consenso brasileiro para diagnóstico e manejo de pacientes com SHUa.

Embora artigos semelhantes tenham sido publicados anteriormente a nível mundial, a população brasileira é única^7^, e essas particularidades, juntamente com a dificuldade de acesso a todos os exames e tratamentos, justificam o desenvolvimento de um documento de consenso nacional.

## Metodologia

### Objetivos do Consenso Brasileiro Para a SHUa

Este documento de consenso foi desenvolvido como parte de uma iniciativa coordenada pelo COMDORA-SBN a fim de padronizar o diagnóstico e o manejo da SHUa no Brasil.

Um painel de especialistas brasileiros desenvolveu este documento com base na revisão da literatura, em dados do Registro Brasileiro de SHUa e em sua própria experiência com esses pacientes. Foi realizada uma reunião em São Paulo nos dias 19 e 20 de agosto de 2023 para definir os pontos-chave do documento. A revisão da literatura foi realizada a partir das seguintes bases de dados: PubMed, Scielo, LILACS (*Latin American Research Review*) e *Cochrane Library*. Os descritores utilizados foram: “Síndrome Hemolítico-Urêmica Atípica” OU “SHUa” E “Diagnóstico” OU “Tratamento”. Os critérios de inclusão utilizados foram artigos publicados até agosto de 2023 em inglês, português ou espanhol.

A qualidade das evidências foi determinada com base na revisão da literatura. Em doenças raras, a obtenção de evidências de alta qualidade é desafiadora devido ao pequeno número de pacientes e à heterogeneidade clínica. Como os estudos controlados e randomizados são escassos, as recomendações foram derivadas de revisões sistemáticas, ensaios clínicos randomizados, diretrizes previamente publicadas, séries de casos, estudos de coorte e dados de registros que refletem dados do mundo real^12^. Além disso, meta-análises de ensaios individuais podem ajudar a abordar essa questão^13^.

A experiência pessoal dos painelistas também foi considerada, especialmente em questões controversas. O sistema GRADE foi usado para classificar a força das recomendações e a qualidade das evidências (Quadro 1)^14,15^.

**Quadro 1:** Nível de recomendação e qualidade das evidências

Nível de recomendação	
1. Recomendação forte	Os benefícios médicos e econômicos são evidentes.
2. Recomendação fraca	Os benefícios médicos e econômicos sugerem algum benefício. As evidências não são suficientes para uma recomendação forte.
Qualidade das evidências	
A	Evidências de alta qualidade: Evidências provenientes de uma meta-análise de ensaios clínicos randomizados ou de pelo menos um ou mais ensaios clínicos randomizados.
B	Evidências de qualidade moderada: Evidências provenientes de um estudo controlado randomizado com uma limitação grave ou de estudos observacionais em larga escala.
C	Evidências de baixa qualidade ou qualidade muito baixa: Evidências provenientes de estudos observacionais em pequena escala ou estudos descritivos não experimentais, como estudos comparativos, estudos de correlação, estudos de caso-controle ou opiniões de especialistas.

Adaptado de Cheong *et al*., 2016^15^.

## Critérios Diagnósticos da SHUA

### Reconhecimento da Microangiopatia Trombótica (MAT)

O diagnóstico baseia-se nas características histopatológicas, mas a biópsia renal costuma ser um desafio devido à trombocitopenia e à apresentação clínica grave^16^. Os achados histopatológicos são complexos e diversos e podem ser resumidos conforme mostra a Figura 1. A suspeita de SHUa começa com a tríade de MAT: anemia hemolítica microangiopática (AHMA), trombocitopenia (absoluta ou sinais de consumo progressivo de plaquetas) e lesão de órgãos (rins, coração, cérebro, trato gastrointestinal e outros)^16^. O envolvimento renal, observado em toda a população brasileira com SHUa, é comum^7^. Essa síndrome pode se manifestar em qualquer idade, independentemente de ser hereditária ou adquirida^7^.

**Figura 1:** Classificação prática das microangiopatias trombóticas. Modificado de Genest *et al*., 2023^17^.

É importante ter em mente que existem algumas condições que podem mimetizar a MAT, como próteses valvares cardíacas ou ocorre no uso de *bypass* cardiopulmonar^18^, crise falciforme em pacientes com anemia falciforme e até mesmo êmbolos de neoplasia metastática. Essas condições também podem se manifestar com AHMA, que são frequentemente associadas a trombocitopenia e disfunção orgânica, embora não sejam classificadas como MAT.

Em 2017, a iniciativa KDIGO (*Kidney Disease Improving Global Outcomes*) listou todas as causas conhecidas de MAT^19^. Tradicionalmente, a MAT é dividida em primária e secundária^19^.

**MAT primária:** As causas primárias de MAT possuem um mecanismo fisiopatológico bem conhecido e um tratamento estabelecido. Classicamente, incluem-se a púrpura trombocitopênica trombótica (PTT) – deficiência grave de uma desintegrina e metaloproteinase com um motivo de trombospondina tipo 1, membro 13 (ADAMTS13, também conhecida como protease de clivagem do fator de von Willebrand) - e a SHUa^20^. Os demais pacientes com MAT são classificados como portadores de MAT secundária.

**MAT secundária:** As causas secundárias de microangiopatia trombótica geralmente ocorrem no contexto de doenças sistêmicas, e a MAT normalmente se resolve com o tratamento ou a remoção da causa subjacente. As causas secundárias clássicas incluem MAT associada à toxina Shiga (ST) produzida por *Escherichia coli* (EC), conhecida como síndrome hemolítico-urêmica típica (SHU) ou SHU-STEC, SHU associada a outras infecções, como SHU relacionada a *Streptococcus pneumoniae* (SHU relacionada a *Sp*), MAT relacionada à gestação, transplantes de órgãos sólidos (especialmente rins) e de células-tronco hematopoiéticas, malignidades, doenças autoimunes, medicamentos e hipertensão maligna^16,20^. Elas são mais frequentes do que a MAT primária. Uma análise de 500 pacientes de quatro centros franceses revelou que 94% dos casos foram secundários a gestação (35%), infecção (33%), medicamentos (26%), neoplasias (19%), transplantes (17%), doenças autoimunes (9%), hipertensão maligna (4%) e outros fatores (6%)^20,21^.

O diagnóstico de SHUa é estabelecido somente após a exclusão de outras causas de MAT, como PTT, STEC-SHU^16^ e condições de MAT secundária^19,22^.

No entanto, à medida que nossa compreensão da MAT avança e os mecanismos subjacentes são elucidados, a classificação e a nomenclatura da MAT continuam a evoluir. Um dos esquemas práticos sugeridos por Genest et al.^17^ oferece uma nova abordagem de classificação da MAT. No presente documento, os autores modificaram a proposta de Genest et al.^17^ e classificam a MAT nas seguintes categorias:

1) PTT, congênita ou adquirida; 2) SHUa, uma MAT mediada por complemento causada por variantes em genes associados ao complemento (congênita) ou por desregulação do complemento mediada por anticorpos, como autoanticorpos anti-CFH (autoimune); 3) MAT associada a variantes em genes não relacionados ao complemento, como os envolvidos no sistema de coagulação (por exemplo, *DGKE*, *THBD*) ou a defeitos metabólicos, como distúrbios no metabolismo da cobalamina; 4) MAT associada a infecções, incluindo STEC-SHU e outras; e 5) MAT secundária a doenças sistêmicas ou à exposição a medicamentos. Essa classificação revisada está ilustrada na Figura 1 ^17^.

### Determinação da Etiologia da MAT

Uma vez identificada a MAT, o desafio é estabelecer a causa correta para iniciar imediatamente um tratamento personalizado. A anamnese, o exame físico e o histórico de saúde familiar auxiliam na identificação da etiologia da MAT. Um histórico familiar positivo aumenta a suspeita de uma doença de origem genética. Além disso, o reconhecimento de sintomas semelhantes aos observados na STEC-SHU ajuda a determinar a etiologia^7^.

O próximo passo é avaliar a gravidade da lesão aos órgãos, que determina a apresentação clínica e é crucial para o manejo de situações de risco à vida^8,11^. Uma abordagem sistemática para identificar a causa subjacente é essencial para reavaliar a terapia direcionada^11^.

### Critérios Diagnósticos de SHUa

O diagnóstico de SHUa é clínico e é estabelecido após a exclusão de outras causas de MAT, como PTT e STEC-SHU, e condições de MAT secundária^16^. As recomendações para testes de diagnóstico são mostradas na Quadro 2.

**Quadro 2:** Recomendações para testes diagnósticos

Testes diagnósticos	
**Confirmação de MAT**	Avaliação bioquímica
	Exame hematológico: hemoglobina, plaquetas e reticulócitos
	LDH sérico
	Haptoglobina sérica
	Esfregaço de sangue periférico (detectar a presença de esquizócitos)
	Coombs indireto/Combs direto
	TP/TTPa/fibrinogênio
**Exames complementares e outros exames/Etiologia**	**Detectar STEC**
	Cultura de fezes ou *swab* retal
	PCR para genes de virulência de STEC nas fezes
	Sorologia: soro (E. coli) e anticorpos contra Yersinia (anticorpos anti-LPS para sorotipos prevalentes)
	**Detectar deficiência de ADAMTS-13**
	Atividade da protease de Von Willebrand
	**Testar causas subjacentes (causas secundárias)**
	Homocisteína plasmática (níveis aumentados são observados em distúrbios de cobalamina)
	Sorologia para HIV, culturas pulmonares, influenza
	ANA / anti dsDNA (Farr)/ anticorpos anticentrômero / anticorpos antifosfolipídios (anticardiolipina IgG e IgM, anti-B / anticoagulante lúpico)
	Hemoculturas
	Teste de gravidez
	Radiografia de tórax
	Fator H; Anticorpos contra o Fator I
	CH50 sérico
	CAM sérico (C5b-9)
	Níveis séricos de C3, C4; índice C3d/C3
	Expressão de CD46 em leucócitos (leucócitos poli ou mononucleares utilizando um teste FACS)
	Níveis sanguíneos do Fator B, Fator Bb, C3 convertase, atividade do Fator H, anticorpos contra o Fator I, outros fatores do complemento e AP50
**Testes genéticos/ Etiologia**	Fator H do complemento (gene *CFH*)
	Fator I do complemento (gene *CFI*)
	Proteína cofator de membrana (gene *MCP*)
	Fator B do complemento (gene *CFB*)
	Complemento C3 (gene *C3*)
	Proteínas relacionadas ao fator H do complemento (genes *CFHR*)
	Gene híbrido *CFH-CFHR*
	Variantes *DGKE* (crianças menores de 2 anos de idade, especialmente se a síndrome nefrótica estiver associada)
	Trombomodulina (gene *THBD*)
	Gene *ADAMTS13*: se indicado (PTT)
	Gene *MMACHC*: se indicado para excluir defeito na deficiência de cobalamina (especialmente em pacientes com menos de 18 anos)
	Outros genes do complemento, se indicado

Abreviaturas – MAT: microangiopatia trombótica; LDH: lactato desidrogenase; TP: tempo de protrombina; TTPa: tempo de tromboplastina parcial ativada; STEC: Escherichia coli produtora de toxina Shiga; PCR: reação em cadeia da polimerase; ANA: anticorpo antinuclear; CH50: medição do complemento hemolítico de 50%; MAC: complexo de ataque à membrana; AP50: via alternativa do complemento hemolítico.

Os critérios diagnósticos recomendados são apresentados na Quadro 3 e na Figura 1.

**Quadro 3:** Critérios diagnósticos de SHUa

Critérios diagnósticos	
Biópsia renal (quando disponível e na ausência de contraindicações).	Sinais de MAT.
Marcadores de microangiopatia trombótica	LDH elevado, AHMA, trombocitopenia, esquistócitos no esfregaço de sangue periférico, haptoglobina baixa, teste de Coombs direto negativo, bilirrubina indireta elevada.
Atividade da *ADAMTS13*	Resultado normal (superior a 10%).
PCR para toxina Shiga ou cultura de fezes	Resultado negativo.
Nível plasmático de homocisteína	Resultado dentro do intervalo normal. Nota: níveis elevados, especialmente em pacientes com menos de 18 anos, podem estar associados a distúrbios do metabolismo da cobalamina.

Abreviaturas – MAT: microangiopatia trombótica; LDH: lactato desidrogenase; AHMA: anemia hemolítica microangiopática; PCR: reação em cadeia da polimerase.

Suspeita-se de SHUa em pacientes com MAT após a exclusão de causas secundárias, ou seja, atividade de ADAMTS13 acima de 10%, descartando PTT, e testes para STEC-SHU negativos (grau 1B). Sempre que disponível, a ativação do complemento deve ser investigada com base em recursos locais. A medida do C5b-9 plasmático que seria ideal, não é disponível ainda na prática clínica. Os níveis plasmáticos de C3 podem ser avaliados - níveis baixos são encontrados em menos de 20% dos pacientes e níveis normais não excluem a SHUa^23^ (grau 1B). Ainda não há consenso sobre os testes de complemento na SHUa.

Se o teste de atividade da ADAMTS-13 não estiver disponível ou enquanto se aguardam os resultados, o escore PLASMIC é uma ferramenta útil à beira do leito para diagnosticar PTT, possibilitando o tratamento precoce dessa doença letal. A sensibilidade e especificidade de um escore PLASMIC igual ou superior a 6 foram de 0,85 (intervalo de confiança de 0,67–0,94) e 0,89 (intervalo de confiança de 95% de 0,81–0,94)^24^. O escore PLASMIC é mostrado na Tabela 1 ^25^ e o uso de calculadoras online pode ser útil (www.mdcalc.com). Somente 5% dos pacientes com PTT apresentam a pêntade clássica: febre, anemia hemolítica, trombocitopenia, manifestações neurológicas e injúria renal.

**Tabela 1:** Escore PLASMIC

Parâmetros	Pontuação
Contagem de plaquetas <30 x 10^9^/L	1
Hemólise (contagem de reticulócitos >2,5%, haptoglobina indetectável ou bilirrubina indireta >2 mg/dL)	1
Sem câncer ativo	1
Sem histórico de transplante de órgãos sólidos ou de células-tronco	1
VCM <90fL	1
RNI <1,5	1
Creatinina <2 mg/dL	1

Abreviaturas – RNI: razão normalizada internacional; VCM: volume corpuscular médio. Adaptado de Vyas *et al.,* 2023^25^

A interpretação dos escores PLASMIC é a seguinte^25^:

• Total de pontos = 0 a 4 - **baixo** risco de deficiência grave de ADAMTS-13
• Total de pontos= 5 - risco **intermediário** para deficiência grave de ADAMTS-13
• Total de pontos= 6 ou 7 - **alto** risco de deficiência grave de ADAMTS-13

### Questões Especiais no Diagnóstico de SHUa

#### A) Papel da biópsia renal na SHUa

As principais características histopatológicas da SHUa são: edema de células endoteliais, expansão subendotelial devido a edema ou aumento dos componentes da matriz e descolamento da membrana basal, acúmulo de detritos no espaço subendotelial e aumento da expressão do fator de Von Willebrand, que atrai plaquetas e leva à formação de microtrombos - os quais ocluem parcial ou totalmente o lúmen dos vasos na microvasculatura. Essa oclusão leva à destruição mecânica de eritrócitos por estresse de cisalhamento, o que explica a anemia intravascular (hemólise intravascular), a adesão plaquetária com trombocitopenia, os eritrócitos fragmentados (esquistócitos) no sangue periférico e a isquemia tecidual variável no tecido.

A biópsia renal não é obrigatória para o diagnóstico de MAT, já que existe uma correspondência clínica com a tríade AHMA, trombocitopenia e lesão de órgãos (particularmente renal)^26^. No entanto, é recomendada em situações especiais, como disfunção do enxerto renal, em que os achados histológicos podem discriminar entre MAT e rejeição do enxerto, definir a presença de glomerulonefrite subjacente e determinar o índice de cronicidade para manejar as expectativas de tratamento (grau 1B)^26^. As Figuras 2,3,4,5 mostram alguns exemplos de critérios de diagnóstico histológico de MAT.

**Figura 2:** Microangiopatia trombótica aguda, subaguda e crônica nos glomérulos. Aguda: A) Coloração H&E mostrando trombo de fibrina (setas) em alguns tufos glomerulares. Há edema endotelial difuso e recrutamento de leucócitos ao redor das alças capilares glomerulares. B) Coloração de tricrômico de Masson revelando trombo de fibrina (setas) obliterando as alças capilares, juntamente com edema endotelial e permeação de leucócitos nas alças capilares. C) H&E mostrando alças capilares em duplicidade, edema endotelial, fragmentação de hemácias, macrófagos espumosos e alguns trombos de fibrina obliterando o lúmen de vários tufos glomerulares. D) Coloração de tricrômico de Masson com alças capilares congestas, duplicação da membrana basal, edema endotelial e fragmentação de hemácias (esquistócitos - seta). Subaguda: E) H&E mostrando glomérulo com expansão mesangial com matriz vacuolada (mesangiólise - setas - resultante de processo trombótico e reparo vascular). Crônica: F) Coloração de prata metenamina de Jones exibindo um glomérulo com duplicação da membrana basal glomerular, edema endotelial, alças capilares colapsadas com “hiperplasia de podócitos” - asteriscos. G) Coloração de prata metenamina de Jones identificando uma arteríola interlobular com trombo de fibrina obstruindo o lúmen do vaso. A jusante, é visível o glomérulo com alças capilares corrugadas, isquêmicas/anêmicas. H) Coloração PAS revelando glomérulos encolhidos, isquêmicos e anêmicos, com dilatação do espaço urinário e “hiperplasia de podócitos” (resultante de lesão vascular trombótica a montante - asteriscos). I) PAS exibindo glomérulos com alças capilares difusamente duplicadas, células endoteliais edematosas (glomerulares e vasculares) com arteríolas mostrando estreitamento do lúmen vascular (setas).

**Figura 3:** Vasculopatia trombótica aguda, subaguda e crônica. Aguda: A) Coloração de tricrômico de Masson mostrando malha de fibrina aderida ao endotélio vascular e estendendo-se para o lúmen vascular arterial. B) Coloração H&E mostrando o lúmen do vaso arterial com obliteração por um trombo de fibrina que abriga leucócitos, plaquetas e glóbulos vermelhos inteiros e fragmentados. C) Coloração de tricrômico de Masson exibindo artérias com trombos de fibrina (em vermelho) obliterando o lúmen vascular. Subaguda: D) Coloração H&E exibindo um vaso arterial com paredes difusamente edematosas e glóbulos vermelhos fragmentados. E) Coloração H&E de vaso arterial com edema mucoide (claro/azul claro) e obstrução do lúmen vascular. Crônica: F) Coloração PAS indicando um vaso arterial com “lesão em casca de cebola”, característica de dano endotelial/vascular crônico.

**Figura 4:** Dano endotelial de microangiopatia trombótica - análise ultra estrutural – A e B) Seção semifinas corada com Azul de Toluidina mostrando glomérulos com alças capilares edematosas e duplicadas, edema endotelial e estreitamento do lúmen vascular. C a F) Seções ultrafinas analisadas por microscopia eletrônica de transmissão contrastadas com tetróxido de ósmio, acetato de uranila e vermelho de rutênio. C e D) Alças capilares difusamente duplicadas, com expansão da lâmina rara interna por material elétron-lúcido e indício de uma membrana basal recém-formada. E) Alargamento do espaço subendotelial com deposição de tactóides de fibrina (seta). F) Em detalhe, a expansão da lâmina rara interna com material lucente e o apagamento das fenestrações endoteliais (seta) devido à lesão endotelial.

**Figura 5:** Microangiopatia trombótica (perfil imunofenotípico). A) Coloração granular por imunofluorescência para IgM nas paredes e no lúmen de vasos arteriais com lesão vascular trombótica (provável aprisionamento na malha do trombo). B) Coloração granular por imunofluorescência para fibrinogênio nas paredes dos vasos arteriais com lesão vascular trombótica (resultante de dano endotelial e extravasamento de plasma/fibrina). C) Coloração granular por imunofluorescência para C3 nas paredes vasculares e no lúmen vascular (devido à ativação do complemento). D) Imunohistoquímica com deposição do complexo de ataque à membrana (C5b9) no lúmen e na parede vascular. E) Imunohistoquímica com coloração granular grosseira nas paredes dos vasos arteriais e no lúmen vascular (fragmento do complemento C4d produzido pela ativação da via clássica e da lectina com alta estabilidade tecidual). F) Imunohistoquímica para marcador de agregação plaquetária CD61 com coloração nas paredes e no lúmen.

#### B) Papel dos testes genéticos na SHUa

Há uma base genética conhecida para quase dois terços dos casos de SHUa, a maioria dos quais está relacionada a uma mutação inativadora das proteínas que inibem a via alternativa: Fator H (*CFH*), Fator I (*CFI*), proteína cofator de membrana (*MCP* ou CD46), trombomodulina (*THBD*), proteínas relacionadas ao Fator H 1 a 5 (*CFHR1-5*) ou uma mutação de ganho de função dos fatores ativadores dessa via do complemento, *C3* ou Fator B (*CFB*)^23^.

A formação de anticorpos IgG anti-CFH foi encontrada principalmente em pacientes pediátricos e está associada a rearranjos genéticos (grandes deleções homozigóticas) nas proteínas 1 e 3 relacionadas ao CFH (deleção *CFHR1-CFHR3*) em 87% dos casos^19,27,28^.

No Registro Global de SHUa^3^, aproximadamente 40% dos 851 pacientes estudados não apresentaram variante ou variantes de risco identificadas em genes do complemento. Isso pode ser devido a alterações em outros genes do complemento ou de coagulação, conforme demonstrado em um estudo de sequenciamento do exoma realizado em 10 pacientes pediátricos com SHUa^29^. No Brasil, 33,5% dos pacientes submetidos à análise genética revelaram ausência de variantes genéticas^6,7^.

Há uma grande variação entre os grupos e laboratórios que realizam a análise genética da SHUa, sendo o método mais comum um painel de sequenciamento de próxima geração (NGS, por sua sigla em inglês) contendo genes da via alternativa do complemento (*CFH, CFI, CFB, C3, MCP, THBD*). Outros laboratórios também analisam genes de coagulação (*PLG, DGKe*), grandes deleções ou rearranjos de genes relacionados ao Fator H (*CFHR1* a *5*) e genes da via das lectinas (*MASP2*). Ainda não há consenso sobre quais genes devem compor o painel ideal de NGS.

Nesse contexto, os achados do Registro Brasileiro de SHUa coincidem em grande parte com os do Registro Global, revelando uma predominância de variantes de *CHF* em todas as faixas etárias e uma ausência de variantes de *CFI* em pacientes pediátricos^3^. No entanto, uma proporção maior de variantes foi identificada em genes que codificam proteínas relacionadas ao Fator H (*CFRH*) no Brasil em comparação com outras coortes^29,30^. A grande deleção *CFHR1-CFHR3* também foi detectada em uma proporção elevada de pacientes brasileiros. Esse achado sugere que a Amplificação Multiplex de Sondas Dependente de Ligação (MLPA), um padrão ouro para a determinação do número de cópias de DNA, deve ser realizada nesses pacientes, especialmente quando nenhuma variante relacionada à doença (grau 1B) tiver sido detectada pelo NGS^6,7^.

Os pacientes frequentemente apresentam variantes em mais de um gene ou polimorfismos, o que pode demonstrar um efeito aditivo de diversos fatores genéticos. Apesar dos avanços, ainda existem dúvidas sobre a base genética da SHUa, uma vez que a correlação genótipo-fenótipo pode envolver genes modificadores, eventos epigenéticos e fatores ambientais. Alguns portadores assintomáticos possuem alterações genéticas, enquanto outros com doença grave têm resultados inconclusivos de testes genéticos. Embora a análise genética auxilie na compreensão da patogênese, os achados negativos não descartam a SHUa e o diagnóstico depende de marcadores clínicos^16^.

#### C) Sobreposição de variantes genéticas relacionadas à SHUa e outras causas de MAT

Variantes genéticas relacionadas à SHUa já foram descritas em pacientes com STEC-SHU^31^, MAT associada à gestação^32^, doenças autoimunes refratárias ao tratamento^33^, transplante de células hematopoiéticas^34^ e gamopatia monoclonal^35^.

Portanto, se a MAT persistir após o tratamento da doença subjacente ou da MAT secundária, deve-se explorar a possibilidade de SHUa^36^ ou PTT^37^ concomitantes, o que afeta as estratégias terapêuticas e o prognóstico do paciente. Embora um estudo envolvendo 110 pacientes com MAT secundária tenha detectado achados genéticos semelhantes aos da população geral de pacientes com MAT^38^, outros estudos demonstraram que muitos dos pacientes com MAT secundária refratária ao tratamento da doença subjacente responderam ao eculizumabe, que foi utilizado apenas temporariamente, sem recidiva da MAT após a suspensão^39^.

## Manejo da MAT e SHUa

### Tratamento de Suporte

Os cuidados de suporte seguem os princípios de manejo da IRA: abordagem do equilíbrio volêmico/eletrolítico, controle da hipertensão, ajuste de medicamentos nefrotóxicos, início de diálise, se indicado, e garantia de nutrição adequada. A anemia grave (Hb <7g/dL) requer transfusões de sangue, enquanto as transfusões de plaquetas são reservadas para sangramento ativo ou necessidade de procedimentos cirúrgicos. Amostras de sangue para o teste de Coombs direto devem ser coletadas antes de qualquer transfusão. Medidas adicionais de suporte incluem diálise, troca de plasma e plasmaférese/infusão de plasma^40,41^ (grau 1B).

### Tratamento Específico

Antes da era dos inibidores do complemento terminal, o manejo da SHUa com medidas de suporte era considerado ineficaz, com 50% dos pacientes necessitando de diálise crônica e até 25% dos óbitos ocorrendo na fase aguda da doença^42^. Após a aprovação do inibidor do C5, eculizumabe, pela FDA e pela Agência Europeia de Medicamentos em 2011, entre outras agências mundiais, incluindo a Agência Nacional de Vigilância Sanitária (ANVISA), o eculizumabe tornou-se a terapia de primeira linha para essa doença^41^. Na próxima seção, discutiremos as terapias específicas para a SHUa.

### USO dos Inibidores de C5 – a Era Pós-Eculizumabe

Todos os pacientes com SHUa são elegíveis para a terapia com inibidores de C5^18^, recomendada como tratamento de primeira linha (grau 1A). O início durante a fase aguda melhora a recuperação da função renal^19^.

O eculizumabe, um anticorpo monoclonal humanizado recombinante que tem como alvo o fator C5, bloqueia a porção terminal do sistema complemento, impedindo a formação de C5b-9, que danifica as células endoteliais^41^. Estudos prospectivos de dois anos sobre a eficácia e a segurança do eculizumabe demonstraram melhora da hemólise, trombocitopenia e função renal. Pacientes com doença renal em estágio terminal (DRET) tratados com eculizumabe apresentaram um número menor de manifestações extrarrenais e melhora na qualidade de vida^19,41,42,43,44,45^.

Cada frasco-ampola de Soliris^®^ (nome comercial do eculizumabe) da *Alexion Pharmaceuticals* contém 300 mg em 30 mL de solução para infusão intravenosa, com duração mínima de 35 minutos^43,44,45,46^. A dosagem e o cronograma são mostrados na Tabela 2.

**Tabela 2:** Dosagem e cronograma de eculizumabe para pacientes adultos e pediátricos com SHUa

Peso (kg)	Indução	Manutenção (por tempo indeterminado)
≥40	900 mg por semana durante 4 semanas; 1200 mg na 5a semana.	1200 mg por semana a cada 2 semanas.
≥30 a <40	600 mg por semana durante 2 semanas; 900 mg na 3a semana.	900 mg por semana a cada 2 semanas.
≥20 a <30	600 mg por semana durante 2 semanas; 600 mg na 3a semana.	600 mg por semana a cada 2 semanas.
≥10 a <20	600 mg por semana durante 2 semanas; 300 mg na 3a semana.	300 mg por semana a cada 2 semanas.
≥5 a <10	300 mg por semana, durante uma semana; 300 mg na 2a semana.	300 mg por semana a cada 3 semanas.

Os efeitos colaterais deste medicamento estão associados ao aumento da vulnerabilidade a infecções por germes encapsulados, especialmente a *Neisseria meningitidis.* Além do uso de antibióticos profiláticos, a vacinação com a vacina conjugada tetravalente (MenACW135Y) e a vacina meningocócica B são recomendadas a todos os pacientes para proteger contra a maioria dos sorotipos do meningococo (pelo menos 15 dias antes do início da terapia). Outras vacinas também são recomendadas, como a Pn13, Pn23, Hib e influenza (grau 1A)^41, 42, 43, 44, 45, 46, 47^. Recomendamos também a atualização do calendário de vacinação com doses de reforço. Embora o fabricante recomende antibióticos somente por 15 dias após a vacinação, se a vacinação não foi possível anteriormente, recomendamos o uso de antibióticos profiláticos (contra a doença meningocócica) enquanto o paciente estiver sob tratamento com inibidor de C5 (grau 1A).

O ravulizumabe é um inibidor de C5 aprovado recentemente, com uma meia-vida mais longa, que permite que a dose de manutenção seja estendida para uma vez a cada 4 semanas (para pacientes com menos de 20 kg) ou uma vez a cada 8 semanas (para pacientes com mais de 20 kg). A segurança e a eficácia do medicamento em adultos e crianças (acima de 10 kg) foram confirmadas em estudos prospectivos^48,49^.

O Ultomiris^®^ (nome comercial do ravulizumabe) da *Alexion Pharmaceuticals* oferece frascos de 300 mg em 3 mL, 1100 mg em 11 mL e 300 mg em 30 mL. No Brasil, está disponível apenas a opção de 300 mg/3 mL. Após a diluição, a concentração final deve ser de 50 mg/mL. O tratamento compreende uma dose de ataque, seguida por uma fase de manutenção duas semanas depois, administrada por infusão intravenosa de acordo com a Tabela 3. Os pacientes em transição de eculizumabe para ravulizumabe devem receber uma dose de ataque de ravulizumabe 2 semanas após a dose final de eculizumabe, seguida de doses de manutenção a cada 4 ou 8 semanas com base no peso, conforme descrito anteriormente. Quando o ravulizumabe é utilizado, intervalos mais longos entre as infusões melhoram a qualidade de vida ao minimizar as punções e os deslocamentos^48,49^.

**Tabela 3:** Dosagem e cronograma de ravulizumabe para pacientes adultos e pediátricos com SHUa

Peso (kg)	Dose de ataque (mg)	Manutenção* (por tempo indeterminado)	Tempo mínimo de infusão (dose de ataque/dose de manutenção)
≥100	3000	3600 mg a cada 8 semanas	25/30 minutos
≥60 a <100	2700	3300 mg a cada 8 semanas	35/40 minutos
≥40 a <60	2400	3000 mg a cada 8 semanas	45/55 minutos
≥30 a <40	1200	2700 mg a cada 8 semanas	31/65 minutos
≥20 a <30	900	2100 mg a cada 8 semanas	35/75 minutos
≥10 a <20	600	600 mg a cada 4 semanas	45/45 minutos

Nota – *Primeira dose administrada 2 semanas após uma dose de ataque.

O ravulizumabe demonstrou ser mais econômico do que o eculizumabe, com possibilidade de economia adicional se for utilizado um concentrado de 100 mg/mL^50,51^.

Os critérios recomendados de diagnóstico e tratamento são apresentados na Figura 6 ^52^.

**Figura 6:** Abordagem diagnóstica e terapêutica da MAT.

Abreviaturas – MAT: microangiopatia trombótica; PTT: púrpura trombocitopênica trombótica; PFC: plasma fresco congelado; PCR: reação em cadeia da polimerase; STEC: Escherichia coli produtora de toxina shiga; SHUa: síndrome hemolítico-urêmica atípica; SHU: síndrome hemolítico-urêmica. Adaptado de Claes *et al.,* 2018^51^.

### Recomendação de Monitoramento

Estudos indicam que o monitoramento do bloqueio do complemento pelo eculizumabe com níveis de CH50 pode ajustar os intervalos de infusão para pacientes sem recidiva da doença. Jodele et al.^53^ constataram que os níveis séricos de eculizumabe se correlacionaram com o CH50 em 365 amostras pareadas de 18 pacientes submetidos a transplante de medula óssea, observando que níveis sanguíneos acima de 99 μg/mL suprimiram o CH50^53^.

Recomenda-se o monitoramento do bloqueio do complemento por meio da inibição do CH50 para o eculizumabe (grau 1A). Para o ravulizumabe, a confiabilidade do CH50 não foi comprovada; portanto, aconselha-se o monitoramento clínico associado à medição do nível sérico do medicamento (grau 1A).

### Espaçamento de Doses

Ardissino et al.^54^ propuseram que o espaçamento entre as doses deve ser monitorado para pacientes que mantêm CH50 inferior a 30% sem recidiva da doença e/ou lesão de órgãos. Eles sugeriram que uma dose de 0,75 mg/kg/dia de eculizumabe mantém o bloqueio do complemento por 4 semanas. Volokhina et al.^55^ avaliaram 11 pacientes com SHUa e o espaçamento de seus tratamentos. Com uma manutenção de 1200 mg em intervalos de 4-5 semanas, 80% apresentaram níveis séricos de eculizumabe superiores a 50 μg/mL. Todos os pacientes com níveis > 50 μg/mL apresentaram bloqueio completo do sistema complemento (CH50 inferior a 10%)^55^.

Pode ser viável individualizar o tratamento com níveis séricos de eculizumabe entre 50–100 μg/mL e monitorar o bloqueio do complemento por meio de CH50^56^. Gatault et al.^57^ analisaram 7 pacientes que utilizaram eculizumabe e constataram que aqueles abaixo de 90 kg tinham intervalos de dosagem de 4 semanas, enquanto aqueles abaixo de 70 kg tinham intervalos de dosagem de até 6 semanas^57^.

O espaçamento entre as doses deve ser ajustado de acordo com o perfil do paciente, comorbidades, adesão ao tratamento e dosagem disponível de CH50 e/ou eculizumabe sérico (grau 1B). Para o ravulizumabe, não há estudos que recomendem o espaçamento de doses além das indicações de SHUa no rótulo não, usar bula.

Na prática clínica brasileira, devido aos desafios na medição dos níveis séricos de medicamentos à base de anti-C5, esse consenso recomenda que o intervalo entre as doses não seja modificado até 3 meses do início da terapia, após a melhora dos parâmetros hematológicos, renais e sistêmicos, na ausência de sinais de atividade da doença. A avaliação do paciente deve considerar comorbidades, função renal, idade, adesão ao tratamento, comprometimento e análise genética disponível (grau 2A). O monitoramento do CH50 é essencial e, na sua ausência, o espaçamento entre as doses não é recomendado (grau 1B). Além disso, o espaçamento das doses não é recomendado para pacientes transplantados renais (grau 1A). Consulte a Quadro 4 para os critérios de intervalo entre doses de eculizumabe.

**Quadro 4:** Parâmetros a serem verificados para o espaçamento entre doses de eculizumabe

Considerações envolvidas no manejo do espaçamento de doses do eculizumabe	
Avaliação inicial	Avaliar a resposta clínica do paciente ao eculizumabe, incluindo melhora nos parâmetros hematológicos, renais e sistêmicos.
Monitoramento	Monitorar regularmente a atividade do complemento por meio dos níveis de CH50 e/ou dos níveis séricos de eculizumabe.
Cronograma de avaliação	Avaliar o espaçamento entre as doses somente após pelo menos 3 meses do início do tratamento, desde que haja melhora nos parâmetros clínicos e ausência de atividade da doença.
Avaliação do perfil do paciente	Considerar o perfil do paciente, incluindo comorbidades, função renal, idade, adesão e comprometimento com a terapia.
Evitar em pacientes transplantados renais	Os autores deste consenso não recomendam o espaçamento de doses para pacientes transplantados renais devido ao risco potencial de recidiva da doença.
Análise genética	Se disponível, o resultado do teste genético pode ajudar a predizer a possibilidade de recidiva e sua gravidade.
Acompanhamento	Reavaliar continuamente a resposta do paciente ao espaçamento entre as doses.
Orientação ao paciente	Orientar o paciente sobre a lógica por trás das decisões de espaçamento de dose de modo a garantir sua compreensão e cooperação.

**Sugestão de manejo do espaçamento de doses do eculizumabe**: O CH50 deve ser medido no dia anterior à dose subsequente. Se o CH50 estiver abaixo de 30%, a dose deve ser espaçada em 3 semanas, com a infusão sendo administrada normalmente na terceira semana. Se o paciente mantiver resultados normais de exames (marcadores de MAT) e estiver assintomático, o CH50 deve ser medido no dia anterior à dose subsequente (infusão da terceira semana). Se o CH50 estiver abaixo de 30%, o intervalo pode ser estendido até a quarta semana. Embora alguns estudos sugiram um espaçamento de até 6 semanas com base em fatores e peso do paciente, este consenso não recomenda intervalos superiores a 4 semanas (grau 2A).

Para o ravulizumabe, o fabricante recomenda considerar a interrupção do tratamento com base na observação médica e no perfil do paciente após 6 meses de tratamento, na ausência de atividade da doença. Um estudo sobre pacientes com hemoglobinúria paroxística noturna revelou eficácia na avaliação dos níveis séricos e intervalos de infusão mais longos, de até 10 semanas, reduzindo os custos do tratamento em 37%^58^.

### Descontinuação da Terapia

O elevado custo da terapia, o risco de efeitos colaterais potencialmente graves (aumento do risco de infecção meningocócica) e as infusões intravenosas quinzenais na fase de manutenção motivaram estudos sobre a descontinuação do tratamento com eculizumabe^59,60^.

Muitos estudos observacionais sobre a descontinuação do eculizumabe surgiram na última década. Uma coorte italiana de 16 pacientes com SHUa que descontinuaram o uso do medicamento relatou que 31,2% apresentaram recidiva dentro de 180 dias, e três deles possuíam uma variante de *CFH* ^29,61^. Em uma coorte francesa, 31% dos 38 pacientes apresentaram recidiva em um período de 22 meses após a interrupção da terapia, com a mutação no gene *CFH* correlacionada a manifestações mais graves e recidiva precoce^30^.

Um estudo de coorte holandês sobre o uso restrito de eculizumabe em 20 pacientes com SHUa observou uma taxa de recidiva de 25% ao longo de 1460 dias^62^. Os pesquisadores desenvolveram uma ferramenta matemática para dosagem e espaçamento individualizados de eculizumabe durante a fase de manutenção, orientados pelo monitoramento terapêutico de medicamentos. Com essa abordagem, foram alcançados desfechos terapêuticos e custo-benefício equivalentes, reduzindo os custos da terapia em até 13%^62,63^. Além disso, o início precoce do eculizumabe (dentro de 3 meses) em pacientes com SHUa com envolvimento renal nativo gerou uma taxa de recidiva de 19%, com economia de custos de até 30%^64^.

O primeiro estudo de coorte prospectivo foi publicado por Fakhouri et al*.* ^65^ em 2021 e envolveu 55 pacientes de diferentes centros franceses. A taxa de recidiva foi de 23% e somente 3 pacientes realizaram transplante renal^65^.

Em uma revisão sistemática, Macia et al.^66^ analisaram casos publicados, dados não publicados, estudos clínicos e dados do Registro Global de SHUa. Episódios de recidiva foram encontrados em 4 (66,6%) dos 6 pacientes em relatos de casos não publicados e em 16 (30,7%) dos 52 pacientes em relatos de casos publicados. Em estudos clínicos, a recidiva ocorreu em 12 (19,6%) de 61 pacientes, dos quais 5 (41,6%) apresentaram uma mutação no *CFH*. Por fim, o registro global mostrou 12 (15,7%) recidivas em 76 pacientes que descontinuaram a terapia com eculizumabe^66^.

Uma coorte brasileira de pacientes com SHUa que tiveram descontinuação não planejada do eculizumabe constatou uma incidência cumulativa de recidiva de 58% em quase 400 dias de acompanhamento. Foram incluídos pacientes com rim nativo, receptores de transplante e pacientes em diálise^67,68^.

Embora não haja na literatura diretrizes definitivas sobre a descontinuação da terapia e o momento apropriado para fazê-lo, este consenso recomenda a descontinuação planejada caso estejam disponíveis testes genéticos, a avaliação de componentes do sistema complemento (por exemplo, CH50 e C5b9) ou o nível terapêutico do medicamento. Além disso, a disponibilidade imediata do medicamento para reintrodução em caso de recidiva é obrigatória^62,63,64,65,68^ (grau 1C).

Recomendamos a tomada de decisão compartilhada entre a equipe médica e o paciente em relação à descontinuação do eculizumabe (grau 1A). Os dados de segurança sobre a descontinuação permanecem inconclusivos para determinar a elegibilidade do paciente e o momento apropriado.

Sempre que possível, recomendamos a avaliação laboratorial dos níveis terapêuticos do medicamento e dos componentes do sistema complemento, pelo menos a dosagem sérica de CH50 (grau 1A). Além disso, recomendamos o acesso imediato a medicamentos para tratar pacientes com recidiva (grau 1A).

## Perspectivas Futuras: Novos Inibidores do Complemento

### Pegcetacoplan

O Pegcetacoplan é um novo inibidor do complemento aprovado pela FDA em 2021 para hemoglobinúria paroxística noturna. Este medicamento se liga ao componente C3 do sistema complemento, impedindo sua clivagem e ativação. A dose recomendada baseia-se no peso e, para adultos, é a administração subcutânea de 1080 mg duas vezes por semana. O medicamento vem sendo estudado para glomerulopatia C3, degeneração macular e anemia hemolítica autoimune, com bons resultados^69^. Ainda não existem estudos para a SHUa, mas, por ser um bloqueador complementar proximal, acredita-se que seja benéfico^69^.

### Iptacopan

O Iptacopan é um potente inibidor do CFB que atua na via alternativa do complemento^70^. Existem alguns estudos avaliando esse medicamento em doenças por desregulação do complemento, como a glomerulopatia C3^71^, demonstrando melhora na proteinúria^72^.

Além disso, um ensaio clínico de fase II está em andamento para avaliar o Iptacopan em pacientes com SHUa, mas ainda não há resultados disponíveis. No entanto, essa pode ser outra possibilidade para esse tratamento.

### Crovalimabe

O crovalimabe (RO7112689 ou SKY59; comercializado pela *Chugai Pharmaceutical*) é um novo anticorpo anti-C5 com tecnologia sequencial de reciclagem de anticorpos monoclonais (SMART) que combina ponto isoelétrico, receptor Fc neonatal e engenharia de afinidade dependente de pH^73^. Isso resulta em uma ligação eficiente do C5, maior captação do crovalimabe ligado ao C5 pelas células endoteliais, eliminação do C5 no endossomo e reciclagem eficiente do crovalimabe mediada pelo receptor Fc neonatal. Além disso, o crovalimabe é altamente solúvel, permitindo pequenos volumes de injeção^73^. O crovalimabe se liga à cadeia - do C5 e impede a clivagem do C5 de tipo selvagem e SNP pela C5 convertase. Dois ensaios clínicos estão em andamento para pacientes com SHUa (NCT04958265 e NCT04861259) e estão recrutando pacientes pediátricos, adolescentes e adultos. Este medicamento apresenta grande potencial para uma boa resposta em pacientes com SHUa^73^.

### Biossimilares do Eculizumabe (Elizaria)

O Elizaria, desenvolvido pela *IBC Generium*, Rússia, é o primeiro biossimilar de eculizumabe registrado mundialmente (Soliris^®^, comercializado pela *Alexion Pharmaceuticals*)^74^. Uma série de análises revelou que a sequência de aminoácidos é idêntica e que as estruturas de ordem superior, modificações pós-traducionais, pureza e variantes do produto são altamente semelhantes entre o Elizaria^®^ DP e o Eculizumab RP, exceto por pequenas diferenças na abundância relativa das variantes de carga e das porções de glicano, que não são consideradas clinicamente significativas^74^. No entanto, devido à experiência limitada com esse medicamento em todo o mundo, este consenso recomenda o uso de inibidores anti-C5 de referência, como eculizumabe ou ravulizumabe, em vez de biossimilares.

### Narsoplimabe

O narsoplimabe é um anticorpo monoclonal humanizado anti-MASP2. A MASP2 é uma serina protease associada à via da manose que se liga à via da lectina do complemento. Acredita-se que a hiperativação da MASP2 estimule a via das lectinas, principalmente em doenças autoimunes, MAT associada ao transplante de medula óssea (TMO) e infecções^75^. Esse medicamento é indicado para a MAT relacionada ao TMO, conforme evidências de um estudo de fase II^76^. Não existem evidências relevantes para uso em pacientes com SHUa.

## Situações Especiais

### Pediátrica

Estabelecer o diagnóstico e a etiologia da MAT em crianças é importante para o manejo imediato da doença. Embora haja uma sobreposição de etiologias de MAT em adultos e crianças, algumas das doenças são mais comuns em crianças, enquanto outras ocorrem somente em crianças^77^.

A principal causa de MAT em crianças é a SHU-STEC, seguida por SHUa e SHU-Sp^78^. Especialmente em crianças com menos de 2 anos de idade, existem condições raras, como PTT congênita (causada por variantes no gene *ADAMTS13*), distúrbios metabólicos de cobalamina (causados por variantes nos genes *MMAHC*, defeitos de cobalamina C ou *MTA*, defeitos de cobalamina G^79^), e distúrbios de coagulação que devem ser descartados antes do diagnóstico de SHUa^77^.

Em neonatos, a asfixia perinatal é um diagnóstico diferencial crítico que pode confirmar a MAT. Anormalidades perinatais (devido a razões fetais, maternas ou placentárias) podem prejudicar a troca gasosa fetal ou neonatal, desencadeando a MAT (AHMA, trombocitopenia e diversas lesões de órgãos, principalmente renais)^80^. O tratamento tardio pode resultar em comprometimento grave dos órgãos, incluindo insuficiência cardíaca, hepática e renal, lesões vasculares e encefalopatia^80^.

Sinais de coagulação intravascular disseminada (CIVD) são críticos em recém-nascidos asfixiados^80^, indicando coagulopatia de consumo devido à isquemia/hipóxia^81^. Os marcadores de asfixia perinatal incluem baixo índice de Apgar, acidose metabólica (detectada precocemente no sangue do cordão umbilical) e falência múltipla de órgãos^82,83^ (grau 1B). Entretanto, a sobreposição clínica entre a SHUa neonatal e a asfixia perinatal dificulta o diagnóstico. A SHUa também pode levar à asfixia e a danos cerebrais em recém-nascidos, dificultando a identificação do evento primário^80,83^. O histórico materno e gestacional, a aparência da placenta, as condições de nascimento, o índice de Apgar e a acidose metabólica precoce são cruciais na prática clínica. Baixos níveis plasmáticos de C3 sugerem hiperativação da via alternativa do complemento. A SHUa é o principal diagnóstico em casos de recidiva de MAT^81^, envolvimento neurológico mais grave^80^ e doença acelerada e não consumptiva^83^.

Os médicos devem estar atentos ao desenvolvimento de MAT em recém-nascidos asfixiados, iniciando o tratamento adequado para reverter o quadro da doença. No entanto, se a MAT persistir, considerar o diagnóstico de SHUa neonatal (grau 1B).

### Manifestações Clínicas e Particularidades da Terapia de SHUa na Pediatria

Crianças apresentam níveis significativamente mais baixos de hemoglobina e plaquetas, além de níveis mais elevados de LDH em comparação com adultos^7,84^, indicando um efeito hemolítico potencialmente mais grave na infância. Além disso, as crianças apresentam uma taxa de mortalidade mais alta do que os adultos^7,83^.

O anticorpo monoclonal (mAb) anti-C5 eculizumabe é a terapia de primeira linha para a SHUa em crianças^46,79,82,85,86^ e sua segurança e eficácia foram demonstradas em diversos ensaios clínicos, estudos de coorte e relatos de casos. Especialmente em crianças, o eculizumabe promoveu a remissão da MAT e está frequentemente associado à recuperação total da função renal^46,85,86^.

Se o mAb anti-C5 não estiver imediatamente disponível na emergência, deve-se iniciar a terapia com plasma, incluindo plasmaférese ou infusão de plasma (grau 1B). A escolha do método depende das condições adequadas do serviço, da experiência profissional, do estado clínico e do tamanho da criança. Embora a terapia com plasma não tenha demonstrado eficácia na manutenção da remissão em longo prazo e na promoção da recuperação da função renal, ela pode melhorar transitoriamente a MAT ao fornecer proteínas reguladoras do complemento. No caso da plasmaférese, é possível remover anticorpos anti-CFH^87^.

No entanto, é importante enfatizar a morbidade associada a esse procedimento, especialmente em crianças, relacionada a complicações do cateterismo venoso central e hipervolemia^88^.

A hidroxicobalamina pode ser administrada em caso de emergência, enquanto os resultados dos exames não estão disponíveis. Embora os distúrbios de cobalamina que levam à MAT sejam raros, são tratáveis e não há eventos adversos graves^83^.

Atualmente, outros bloqueadores anti-C5 têm sido estudados em crianças. O ravulizumabe já está aprovado e existem ensaios clínicos pediátricos que demonstram sua eficácia e segurança^89^. Outras opções estão atualmente sob investigação, com posologia aprimorada e a possibilidade de administração subcutânea (crovalimabe) ou oral (iptacoplan).

### Gestação

A MAT associada à gestação é um distúrbio raro com uma incidência estimada de aproximadamente 1 em 25.000 gestações e está associada a morbidade e mortalidade perinatal e materna significativas^90^.

A gravidez e o período pós-parto há muito tempo são reconhecidos como condições de alto risco para a MAT. Existem três principais diagnósticos diferenciais para a MAT associada à gestação: (1) PE / síndrome HELLP (pré-eclâmpsia / hemólise, elevação da função hepática, baixa contagem de plaquetas); (2) PTT; e (3) SHUa. A gestação é um conhecido fator desencadeante para PTT e SHUa, e a presença desses distúrbios aumenta o risco de PE/síndrome HELLP.

Para os marcadores de MAT, alguns especialistas propõem um limite de contagem de plaquetas mais baixo para o diagnóstico clínico, considerando que, em uma gestação normal, há uma redução de plaquetas. Aproximadamente 10% das gestações sem complicações apresentam contagem de plaquetas inferior a 150.000/mm^3^ no momento do parto. Portanto, um limite de 100.000/mm^3^ parece ser adequado para o diagnóstico de MAT associado à gestação^91^. Outros parâmetros, como anemia, elevação de LDH, redução da haptoglobina, presença de esquizócitos e lesão de órgãos, estão em conformidade com as recomendações para outras formas de MAT.

A IRA é frequentemente encontrada na maioria dos tipos de MAT associada à gestação, exceto na PTT. Embora não exista uma definição universalmente aceita de IRA durante a gravidez, as diversas definições disponíveis referem-se às diretrizes do KDIGO^19^. Outras publicações baseiam-se em uma creatinina sérica acima de 0,90 mmol/L e/ou um aumento de 0,25% em relação ao valor basal^92^.

### SHUa na Gestação

A gravidez é uma condição de aumento da atividade de todas as vias do sistema complemento, incluindo as vias clássica, das lectinas e alternativa. O objetivo é limpar a circulação materna de complexos imunes e, por outro lado, de proteínas reguladoras para o controle do complemento (principalmente MCP e CD59). Além disso, estudos identificaram variantes em genes do sistema complemento em mais de 50% dos casos de MAT associada à gestação^93^.

A SHUa, a forma mais rara de MAT na gravidez, geralmente surge no final do terceiro trimestre ou no pós-parto. Casos fora desses períodos complicam o diagnóstico diferencial com PE/síndrome HELLP^91,94^. O comprometimento renal é comum, enquanto a contagem de plaquetas normalmente não é reduzida de forma crítica, e o envolvimento neurológico, ao contrário da PTT, é pouco frequente^91,94,95^.

Atualmente, o tratamento recomendado é um inibidor de C5 (grau 1B). Sem esse tratamento, os desfechos renais são preocupantes, com 76% dos pacientes evoluindo para doença renal em estágio terminal (DRET), apesar de receberem plasmaférese^93.94^. Outro estudo demonstrou um risco de 50% de DRET em mulheres grávidas com SHUa, independentemente de terem sido submetidas à plasmaférese ou não^94^.

Apesar do alto custo do medicamento, ele geralmente não excede o custo do tratamento em unidades de terapia intensiva, plasmaférese, hemodiálise, provável insuficiência renal e transplante^96^. Os mAb anti-C5 podem atravessar a placenta, mas dados limitados ao número de gestações expostas ao eculizumabe (menos de 300 desfechos de gravidez) indicam que não há aumento do risco de malformações fetais ou toxicidade fetal-neonatal^96,97^. Nenhum estudo clínico controlado foi realizado para avaliar a eficácia do anti-C5 na SHUa associada à gestação. Apesar disso, mais de 35 casos foram relatados na literatura em que o eculizumabe foi administrado durante ou após a gestação, com aproximadamente 90% apresentando resposta hematológica e remissão da doença renal^97^.

A duração do tratamento é indeterminada, e a descontinuação do tratamento com anti-C5 deve ser personalizada. As variantes em genes do complemento aumentam o risco de recidiva. O bloqueio do complemento terminal deve ser monitorado, uma vez que a gestação pode exigir dose/frequência mais elevadas devido a alterações de volume, aumento da síntese de C5 ou proteinúria. Apesar do eculizumabe, um histórico prévio de SHUa eleva o risco de recidiva em gestações subsequentes, exigindo monitoramento rigoroso^93^.

De acordo com a bula, o ravulizumabe é classificado como Categoria C durante a gravidez (gestantes não devem usar esse medicamento sem orientação médica). Não há dados clínicos sobre a exposição durante a gestação. Entretanto, estudos recentes relatam a eficácia e a segurança do ravulizumabe na SHUa pós-parto^98^.

## Transplante

### Investigação Pré-Transplante

Pacientes com DRC estágio 5 de causa desconhecida, casos pós-gestação, nefrite lúpica, histologia de MAT e hipertensão maligna devem ser considerados casos potenciais de SHUa. A avaliação pré-transplante deve incluir hemograma com esquizócitos, LDH, teste de Coombs, haptoglobina, autoanticorpos e níveis de complemento (C3 e C4)^99^.

Se houver probabilidade de SHUa e a hemólise for evidente (SHUa ativa), deve-se considerar um curso de 6 meses de mAb anti-C5 antes do transplante para avaliar a possível recuperação da função renal^99^ (grau 1C).

### Análise Genética em Transplantes

A análise genética de todos os genes potencialmente relacionados ajuda as equipes médicas e os pacientes a desenvolverem estratégias para prevenir a recidiva de SHUa pós-transplante^100^ (grau 1C).

O risco de uma recidiva de SHUa no enxerto renal se correlaciona com o tipo de variante genética. O transplante renal em pacientes com SHUa e DRET é complexo, com taxas de recidiva de 50-80%^41,42^ resultando em perda do enxerto em até 91,6% dos casos^42,43,98^. Os receptores de transplante apresentam risco de MAT em decorrência de fatores que lesam o endotélio, incluindo medicamentos imunossupressores (inibidores de calcineurina e inibidores de mTOR), lesão de isquemia-reperfusão, rejeição e infecções pós-transplante^101^.

Após os testes genéticos, os pacientes devem ser estratificados em grupos de risco de recidiva (grau 1A), e o melhor regime profilático deve ser abordado antes da cirurgia. Pacientes de alto risco são aqueles com recidiva prévia de transplante, variantes relacionadas à doença no *CFH* ou variantes de ganho de função no *CFB* ou *C3*. Os pacientes de risco moderado apresentam anticorpos anti-fator H, variantes no *CFI*, variantes de significado incerto ou polimorfismos no *CFH*. Pacientes de baixo risco possuem mutações em *MCP*, anticorpos anti-fator H persistentemente negativos ou ausência de mutações/polimorfismos^101^ (Quadro 5)^99^ e é possível observar desfechos do transplante desses pacientes usando eculizumabe, se necessário.

**Quadro 5:** Algoritmo de investigação para SHUa pré-transplante e risco de recidiva

Algoritmo de investigação SHUa pré-transplante		
Pacientes com suspeita	Testes adicionais	Considerar
**Hipertensão maligna pós-parto** **Biópsia revelando nefrite lúpica por MAT**	HMG com esquizócitos DHL teste de Coombs Haptoglobina C3/C4 Autoanticorpos Antifosfolípides **Histórico extra renal** Cardiomiopatia Cerebrovascular Trombose	Análise genética
**Diagnóstico provável**		
**Provável SHUa ativa**(evidência de hemólise)	Considerar eculizumabe por 6 meses	**Sem resposta:** Lista de transplantes (Avaliar risco de recidiva)
**Provável SHUa**(sem evidência de hemólise)	Lista de transplantes (avaliar risco de recidiva)	
**Risco de recidiva pós-transplante**		
**Risco elevado**	**Risco moderado**	**Risco baixo**
– Recidiva após transplante – Variante de inativação no *CFH* – Variante de ganho de função no *CFB* ou *C3*	– Anticorpo antifator H – Variante no gene *CFI* – Variantes de significado incerto (VUS) – Polimorfismos no *CFH*	– Variantes no *MCP* – Anticorpo contra o fator H negativo – Ausência de mutação ou polimorfismo
**Eculizumabe pré-transplante**	**Eculizumabe pré-transplante**	**Monitoramento Transplante sem eculizumabe**
**Em todos os casos de suspeita de SHUa**		
• Evitar o uso de doadores de critérios expandidos • Evitar o transplante na presença de anticorpos contra o doador • Introduzir profilaxia contra citomegalovírus • Evitar o uso de inibidores de mTOR em combinação com inibidores de calcineurina • Evitar doses elevadas de inibidores de calcineurina		

Adaptado de Zuber *et al*. 2013^99^.

O uso de doadores vivos aparentados não é recomendado para pacientes com SHUa devido aos potenciais riscos de variantes do doador após a nefrectomia (grau 1B). Ao considerar um doador aparentado, a análise genética deve garantir a ausência de variantes do gene do complemento. É fundamental discutir os riscos de SHUa pós-nefrectomia com o potencial doador (grau 1B).

Além disso, recomenda-se evitar o uso de inibidores de mTOR com inibidores de calcineurina, altas doses de inibidores de calcineurina, anticorpos contra o doador no transplante, doadores de critérios expandidos e tempos prolongados de isquemia fria (grau 1B). Essas estratégias visam mitigar o estresse endotelial do enxerto, reduzir a lesão de isquemia-reperfusão e, possivelmente, diminuir a ativação da via alternativa do complemento.

### Diagnóstico de SHUa Pós-Transplante

O diagnóstico de SHUa pós-transplante é semelhante ao do contexto geral. No entanto, algumas causas secundárias especiais devem ser descartadas, como as induzidas por inibidores de calcineurina/mTOR, bem como a rejeição mediada por anticorpos e doenças autoimunes e virais (Quadro 6)^102^.

**Quadro 6:** Etapas para diagnosticar a síndrome hemolítico-urêmica atípica (SHUa) após o transplante

Anemia hemolítica microangiopática (AHMA)	
**ADAMSTS13**	> 5% descarta deficiência grave
**Descontinuação ou redução da dose de inibidores de calcineurina e/ou inibidores de mTOR**	Microangiopatia persistente sugere SHUa
**Exclusão de infecções virais(HIV; HTLV I/II; hepatite B; hepatite C; citomegalovírus; Epstein-Barr)**	
**Exclusão de infecções bacterianas(hemocultura, cultura de urina, cultura de fezes)**	
**Exclusão de doenças autoimunes(FAN, antiDNAn, c-ANCA, p-ANCA, fator reumatoide)**	
**Histologia sugestiva de microangiopatia**	
**Exclusão de rejeição mediada por anticorpos**	Teste negativo para C4d na biópsia e ausência de anticorpos do doador
**Diagnóstico de SHUa**	
**Estudo complementar com análise genética**	

Adaptado de Andrade *et al*., 2017^102^.

Recomenda-se a realização de exames laboratoriais diários até que sejam obtidos parâmetros hematológicos normais e a função renal melhore. Os testes de anemia hemolítica incluem hemograma, contagem de plaquetas, esfregaço de sangue periférico (para esquistócitos), LDH e haptoglobina. O monitoramento da função renal envolve medições da creatinina sérica e da relação proteína/creatinina urinária (grau 1B).

### Recomendações de Tratamento Após o Transplante

O eculizumabe é eficaz em casos de SHUa após transplante renal^101,103,104,105^. O ravulizumabe também é eficaz e seguro em pacientes transplantados, conforme relatos de casos. A dose recomendada desses medicamentos para pacientes transplantados renais é a mesma indicada para outros pacientes. A imunossupressão com inibidores de calcineurina é recomendada com monitoramento cuidadoso para evitar a superexposição (grau 1B), enquanto os inibidores de mTOR devem ser evitados em pacientes com SHUa submetidos a transplante renal (grau 2B). O belatacept de longa duração pode ser usado para evitar os inibidores de calcineurina, mas esse medicamento não está regularmente disponível no Brasil.

## Conclusão

O grupo de especialistas do COMDORA-SBN fornece recomendações para o diagnóstico e tratamento da SHUa na população brasileira. Essas diretrizes têm como objetivo melhorar, e não restringir, as práticas clínicas atuais. Esse consenso será atualizado regularmente com novas informações e dados, conforme necessário.
